# A systematic review of explainable artificial intelligence and cardiac electrophysiological models addressing sports-related sudden cardiac death and arrest in adolescents and young adults

**DOI:** 10.1038/s41746-026-02878-x

**Published:** 2026-06-11

**Authors:** Erik Vanegas Müller, Nicharee Srikijkasemwat, Andrew Gan, Betty Raman, Mirae Harford, Liang He, Abhirup Banerjee, Katja Gehmlich, Paul Leeson, Mauricio Villarroel

**Affiliations:** 1 The Podium Institute for Sports Medicine and Technology, Department of Engineering Science, University of Oxford, Oxford, UK; 2 Institute of Biomedical Engineering, Department of Engineering Science, University of Oxford, Oxford, UK; 3 Department of Cardiovascular Sciences, University of Birmingham, Birmingham, UK; 4 Division of Cardiovascular Medicine, Radcliffe Department of Medicine, and British Heart Foundation Centre of Research Excellence Oxford, University of Oxford, Oxford, UK; 5 Critical Care Research Group, Nuffield Department of Clinical Neurosciences, University of Oxford, Oxford, UK; 6 NIHR Oxford Biomedical Research Centre, University of Oxford, Oxford, UK

**Keywords:** Cardiology, Diseases, Medical research

## Abstract

Sudden Cardiac Death (SCD) is a fatal event occurring within one hour of a witnessed or 24 hours of an unwitnessed Sudden Cardiac Arrest (SCA), being the leading medical cause of death among adolescent and young adult athletes. We examined the epidemiology of sports-related SCD (SrSCD) and SCA (SrSCA) incidence in adolescents and young adults, explainable Artificial Intelligence (xAI) applied to life-threatening arrhythmias, and cardiac electrophysiological models. We systematically searched peer-reviewed studies from eight databases between 2013–2025 (PROSPERO: CRD42024565960), using PROBAST for bias assessment. From 9574 studies, we included 84 (incidence: 16, xAI: 30, modelling: 38). SrSCD incidence ranged from 0.1 to 0.6 per 100,000 participants per year. Gradient-weighted Class Activation Mapping dominated as xAI technique. Cardiac electrophysiological models predominantly focused on cellular and tissue-level electrophysiology. We advocate for standardised SrSCD/SrSCA definitions and integration of epidemiological risk factors with xAI and cardiac modelling frameworks to advance athlete-specific risk stratification.

**Figure:**
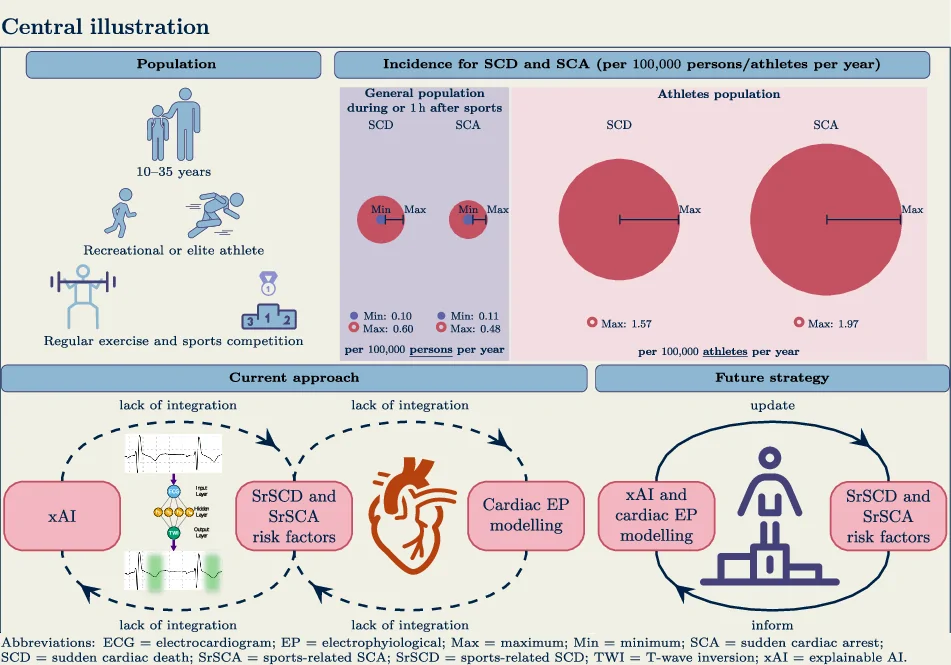

## Introduction

Sudden Cardiac Death (SCD) is a fatal event occurring within one hour of a witnessed or 24 hours of an unwitnessed cardiac arrest. Presumed SCD accounts for approximately half of worldwide cardiac deaths and between 15% and 20% of overall worldwide mortality^1^. Sudden Cardiac Arrest (SCA), the precursor to SCD, involves sudden cardiac rhythm changes to potentially fatal arrhythmias requiring immediate cardiopulmonary resuscitation and defibrillation.

The European Society of Cardiology defines athletes as “individuals of young and adult age, either amateur or professional, who are engaged in exercise training regularly and participate in official sports competition”^2^. The SCD risk is higher among adolescent and young adult athletes (compared to non-athletes), representing the leading medical cause of death in this population, and while exercising^3^. The higher SCD risk in athletes compared to non-athletes persists during exercise^4–7^. The prevailing assumption is that up to one out of 300 athletes may have an arrhythmic substrate predisposing to SCA^3^. Genetic variants (e.g. hypertrophic cardiomyopathy) further increase life-threatening arrhythmia risk, with SCA often being the first clinical manifestation of underlying cardiac electrophysiological disorders^5^. Discerning between exercise-induced physiological adaptations that mimic heart disease and certain cardiac pathologies with the potential for arrhythmia, syncope, and SCD presents a challenging but fundamental clinical problem^8,9^.

Athletes face an elevated SCD risk during exercise and up to 30 minutes after exercise compared to when resting^4,5,10^. Peterson et al.^11^ found that among 331 confirmed SCA/SCD cases in athletes aged 11–29, 74% occurred during exercise and 4% within one hour post-exercise. Petek et al.^12^ reported that 50% of 143 athlete SCAs (mean age 20) occurred during exercise. With 50%^12^–78%^11^ of cases being exercise-related in these two studies, we focus this review specifically on sports-related sudden cardiac death (SrSCD), which is an unexpected, non-traumatic death presumably of cardiac origin during or within one hour following exercise^13^. Its precursor is sports-related sudden cardiac arrest (SrSCA). We hypothesise that exercise and exercise-induced cardiac adaptations play a central role in cardiac events among athletes during or shortly after activity (see Supplementary Information A).

Identifying adolescents (≥10 years) and young adults (≤35 years) with undiagnosed arrhythmias and other electrical disorders (e.g., Wolff-Parkinson-White syndrome) remains challenging^1,3^. Electrocardiograms (ECGs) provide non-invasive risk stratification for adverse arrhythmias. Complex electrophysiological mechanisms, such as myocardial depolarisation and repolarisation, are simplified to one-dimensional signals, such as the QT segment, omitting patterns that may indicate arrhythmic risk. Universal ECG screening in athletes would lead to a surge in ECG evaluations, with insufficient expertise among community-level primary care providers^14^. Artificial Intelligence (AI) can detect morphological ECG changes, classify arrhythmias, predict cardiovascular outcomes, and enable a scalable solution for universal ECG screening. However, AI’s “black box” behaviour requires interpretability and explainability for clinical applications, as a diagnosis without explanation is inherently incomplete. Explainable AI (xAI) delivers human-intelligible explanations for model predictions^15^, thereby enhancing AI’s potential in cardiac health. Beyond classification, xAI explanations can be anchored within cardiac electrophysiological models, grounding the AI in underlying mechanisms.

This systematic review examines three research domains relevant to SrSCD and SrSCA: (1) epidemiological incidence in adolescents and young adults, (2) xAI applied to ECGs for detecting life-threatening arrhythmias, and (3) cardiac electrophysiological models. We synthesise current evidence across these domains and propose future perspectives on how xAI and cardiac electrophysiology models can contribute to the early detection of long-term SrSCD and SrSCA risk in the sports population.

## Results

**Fig. 1:**
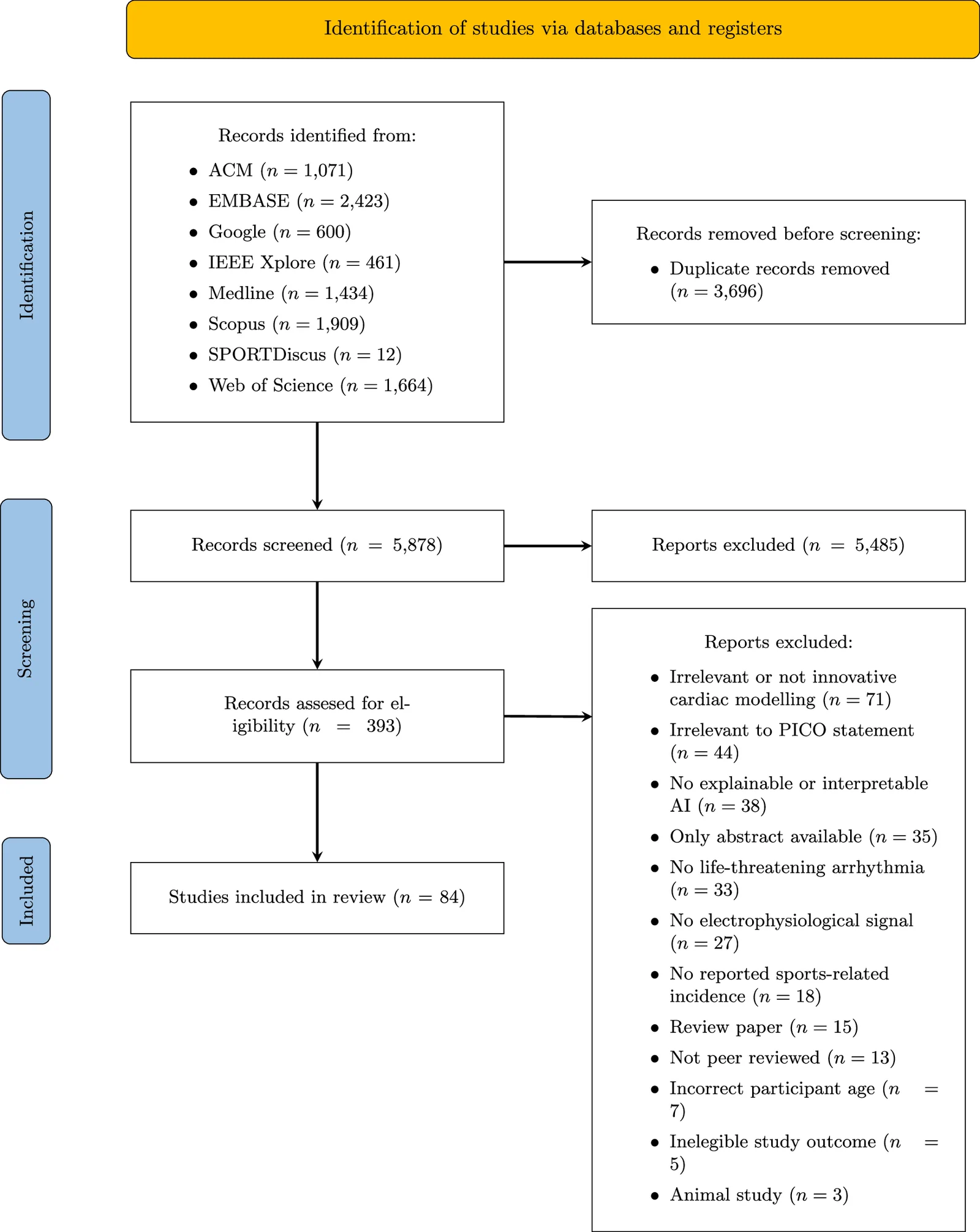
PRISMAdiagram. PRISMA diagram illustrating the sources in identifying studies for this systematic review.

We found 9,574 studies and screened 5,858s after deduplication, of which 393 resulted in full-text screening, and 84 met the inclusion criteria (see Fig. 1). Results comprise three subsections: SrSCD and SrSCA incidences (*n* = 16), xAI applied to life-threatening arrhythmias (*n* = 30), and cardiac electrophysiological modelling (*n* = 38).

### Sports-related sudden cardiac death and arrest incidences

**Fig. 2:**
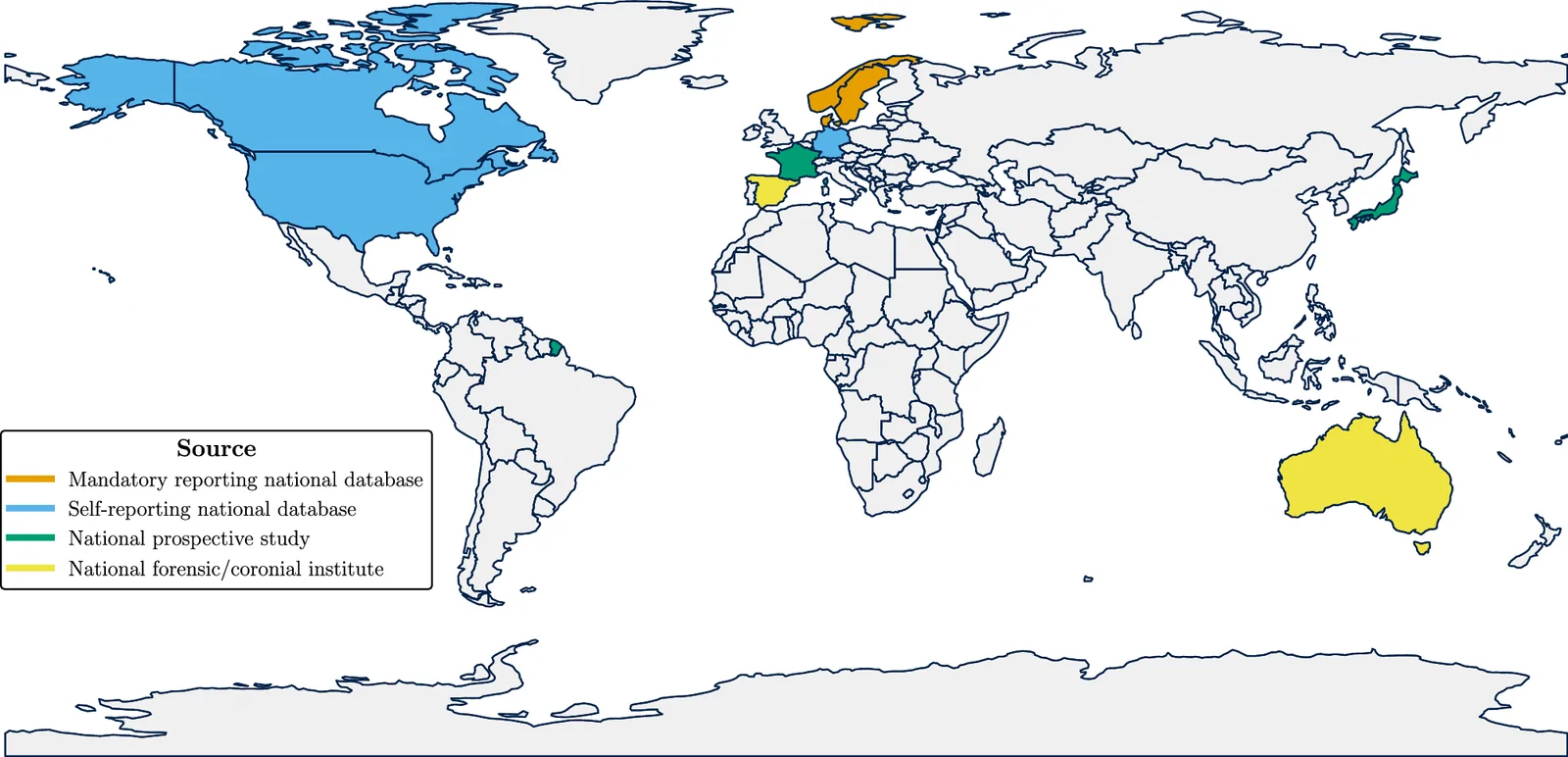
Countries reporting a SrSCD or SrSCA incidence in adolescents and young adults. Only countries with a mandatory national reporting database can report all suspicious SrSCA and SrSCD. Not all countries perform an autopsy on all deaths, which carries the potential for misclassifying the cause of death though all possible deaths are being centrally reported. The predominance of mandatory reporting and forensic databases reflects the concentration of studies in high-income countries, with entire regions such as Africa and Latin America absent from the evidence base.

**Fig. 3:**
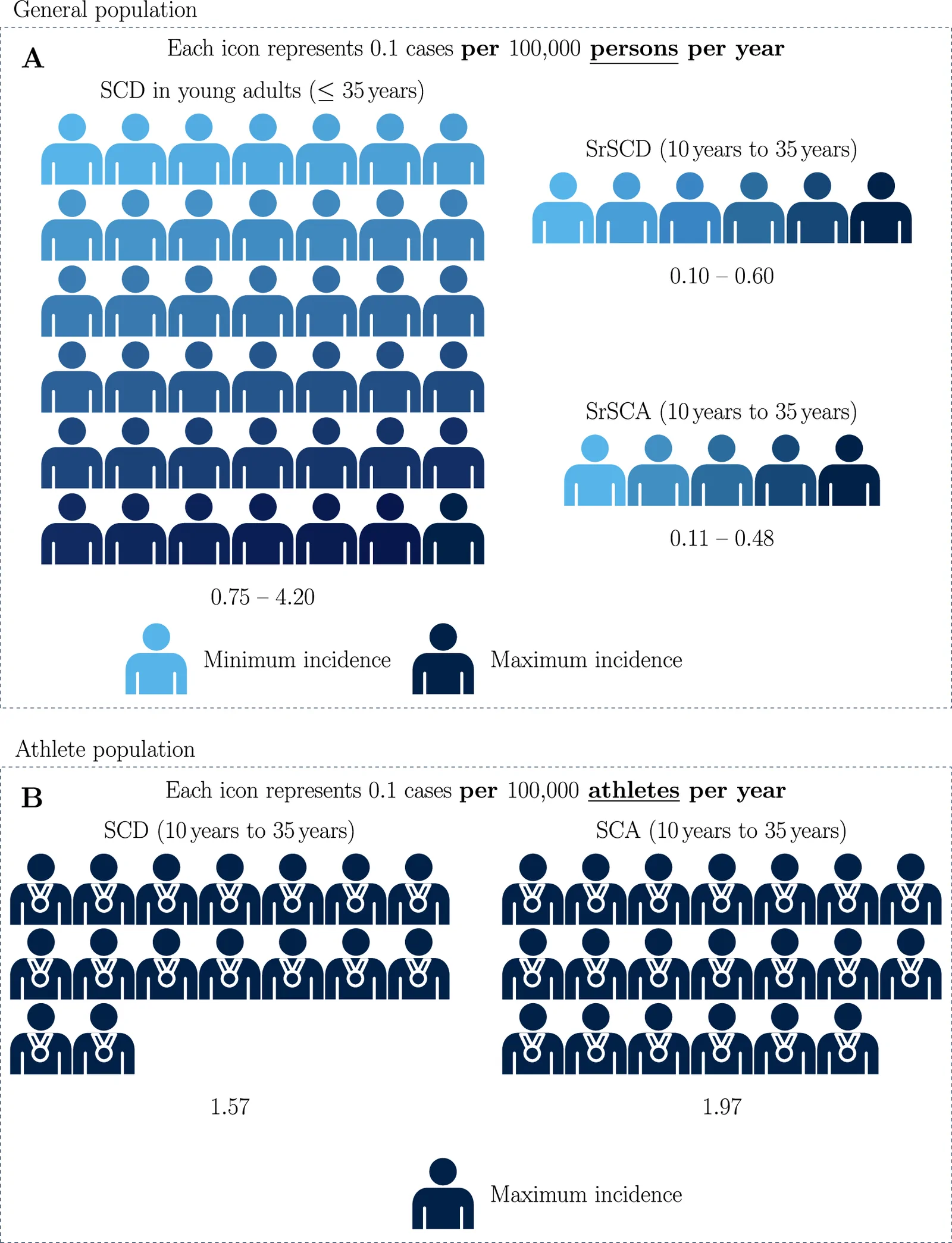
Overview of death and arrest incidences. Incidences of (**A**) SrSCD and SrSCA in the general population and (**B**) SCD and SCA incidences regarding the athlete population (see Table 1). The light blue and dark blue icons represent the rounded minimum and maximum; the icons between them form a colour gradient from light blue to dark blue. Each icon represents 0.1 cases. Note that the population at risk for the general population in (**A**) consists of persons, and athletes for the population in (**B**) (see Table 1 for more information).

Table 1 summarises a selection of the identified incidences which represent the full range and geographical diversity. The SrSCD and SrSCA definitions are that the event occurred during sports or within 1 hour of exercise cessation, and all studies explicitly defined SrSCD or SrSCA. We find studies from Australia, Canada, Denmark, France, Germany, Japan, Norway, Spain, Sweden, and the USA (see Fig. 2). The SrSCD incidence in the general population ranges from 0.10 to 0.60^7^ per 100,000 participants per year, as shown in Fig. 3A, with lower rates for women compared to men. Some studies include only SrSCD in female athletes aged 15–24 years (0.04^16^ per 100,000 participants per year) or in high school athletes from Minnesota (0.68^17^ per 100,000 participants per year). The SrSCA incidence ranges from 0.11^18^ to 0.48^13^ per 100,000 persons per year.

**Table 1:** Selected sport-related sudden cardiac death, sport-related sudden cardiac arrest, and sudden cardiac death and arrest incidences for adolescents and young adults (10–35 years)

Type	Study	Country	Population	Age; sex	Incidence
SrSCD	Marijon et al. ^16^	France	Athletes	15–34 years; 0% male	15–24 years: 0.039, 25–34 years: 0.047 per 100,000 participants per year
SrSCD	Maron et al. ^17^	USA	Athletes	12–18 years; 100% male	0.68 per 100,000 participants per year (*n* = 13)
SrSCD	Risgaard et al. ^19^	Denmark	Athletes	12 – 35 years, 21 ± 8 years; 89% male	Non-competitive: 0.43, competitive: 0.47 per 100,000 athletes per year (n = 9)
SrSCD	Wisten et al. ^7^	Sweden	General	10–35 years; 90% male	Between 0.1 to 0.6 per 100,000 persons per year (*n* = 62)
SrSCA	Bohm et al. ^18^	Germany	Athletes	10–35 years; ---	10–25 years: 0.11, 25–35 years: 0.14 per 100,000 persons per year (n = 90, 29 survived)
SrSCA	Bohm et al. ^13^	Germany and Paris area	Athletes	18–35 years; 95.2% male	0.48 per 100,000 persons per year (n = 147, 56 survived)
SrSCA	Sado et al. ^20^	Japan	Students	Junior high school: 12–15 years; High school/technical college: ≥15 years; 85% male	Junior high school: 0.217 (*n* = 61); high school/ technical college: 0.389 (*n* = 108) per 100,000 students per year
SrSCA	Visanji et al. ^91^	Canada	General	18–34 years (26.3 ± 3.9); 91.3% male (*n* = 21)	0.3 per 100,000 persons per year (*n* = 23), *n* = 16 had a ROSC and *n* = 14 survival to discharge (only of 22)
SCD	Petek et al. ^12^	USA	Athletes	Mean 20 years; 83% male	SCD: 1.57 per 100,000 athletes per year (*n* = 143), 50% happened during exertion.
SCA	Peterson et al. ^11^	USA	Athletes	11–29 years; 83.7% male	SCA: High school: 1.52, NCAA: 1.97 per 100,000 athletes per year (*n* = 331, 158 survived, 78% occurred during or within 1 h of exercise cessation)

We selected these cases to represent the full range of reported incidence and geographical diversity. Only studies reporting calculated incidence rates were included. See Table S1 (SrSCD), Table S2 (SrSCA), and Table S3 (SCD/SCA) in Supplementary Information C for all 16 studies with complete information.

*NCAA* National Collegiate Athletic Association, ROSC Return of Spontaneous Circulation, *SCA* Sudden Cardiac Arrest, *SCD* Sudden Cardiac Death, *SrSCA* Sports-related SCA, *SrSCD* Sport-related SCD.

We include two studies that examine SrSCD and SrSCA cases within the broader context of SCD/SCA in athletes. In Petek et al. ^12^, 50% of recorded SCDs in athletes are SrSCD. In Peterson et al. ^11^, 88% of registered SCA cases in athletes are SrSCA. We mention these studies because these are the only studies, apart from Risgaard et al. ^19^, which calculates SrSCD/SrSCA incidence per athlete rather than per person or participant.

**Fig. 4:**
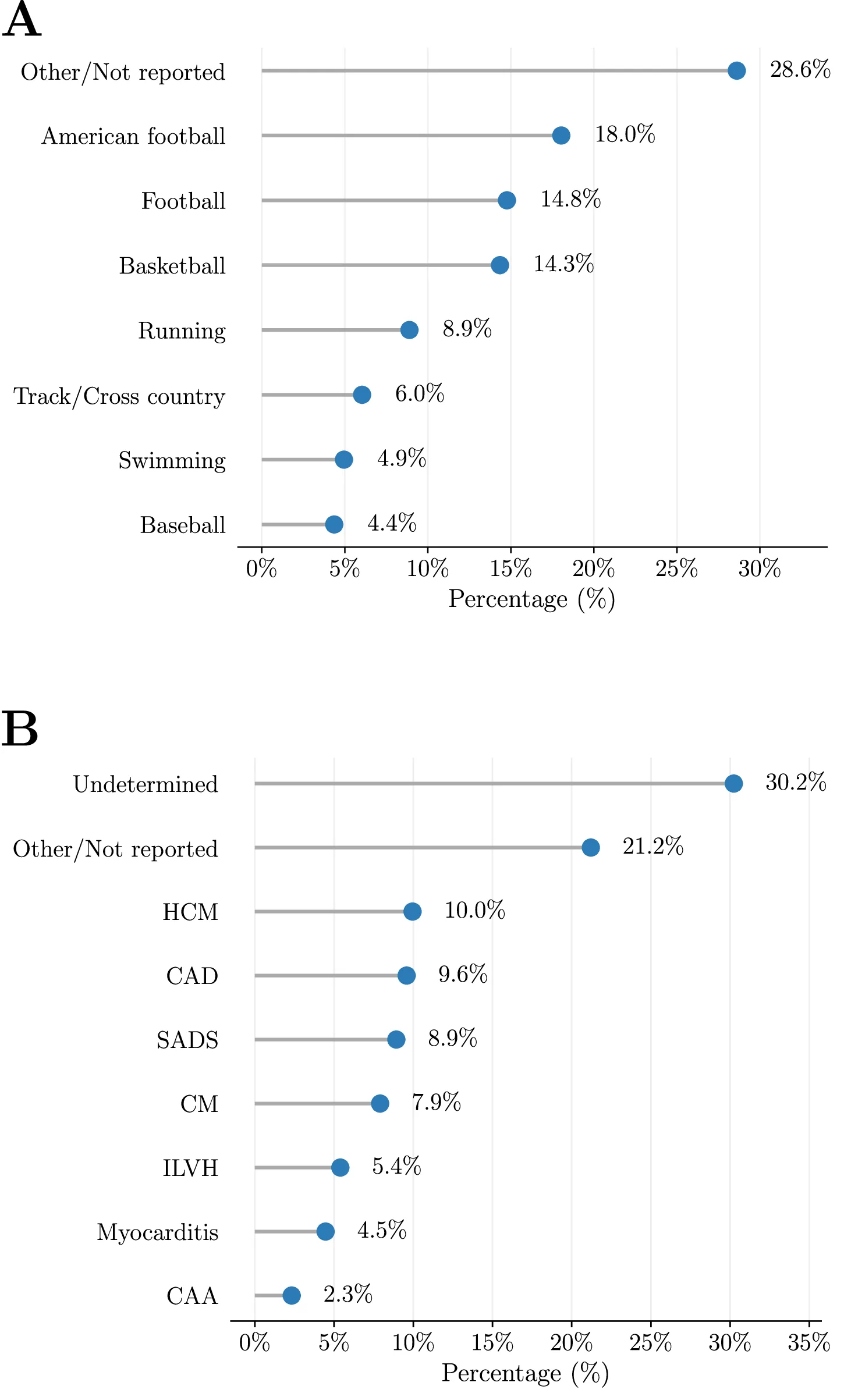
Overview of sport and pathology profiles. Lollipop charts of the (**A**) practised sports and (**B**) the diagnosed underlying pathologies. Subfigure (**A**) presents the practised sports among individuals with a SrSCD or SrSCA in the general population and SCD or SCA in the athlete population (see Table 1). Other sports (28.6%) include volleyball, wrestling, or boxing, for example, or were not reported. Subfigure (**B**) shows the diagnosed underlying pathology in the general population, and SCD or SCA in the athlete population (see Table 1). Other causes of death include pathologies such as valvular heart diseases or ARVC, for example. We included SADS and sudden unexplained death in the SADS category. ARVC, Arrhythmogenic Right Ventricular Cardiomyopathy; CAA, Coronary Artery Anomaly; CAD, Coronary Artery Disease; CM, Cardiomyopathy; HCM, Hypertrophic Cardiomyopathy; ILVH, Idiopathic Left Ventricular Hypertrophy; SADS, Sudden Arrhythmic Death Syndrome.

In Europe, football and running are the predominant sports during SrSCD/SrSCA, whilst basketball and American football are the most common in the USA (see Fig. 4A). The majority of cardiac deaths or arrests have an undetermined cause (see Fig. 4B). Male predominance is consistent across all studies, ranging from 83%^12^ to 100%^17^. Four studies indicate that SCA/SrSCD/SrSCA incidence increases with age, ranging from 20%^16^ to 79%^20^ in adolescents and young adults.

### xAI

**Fig. 5:**
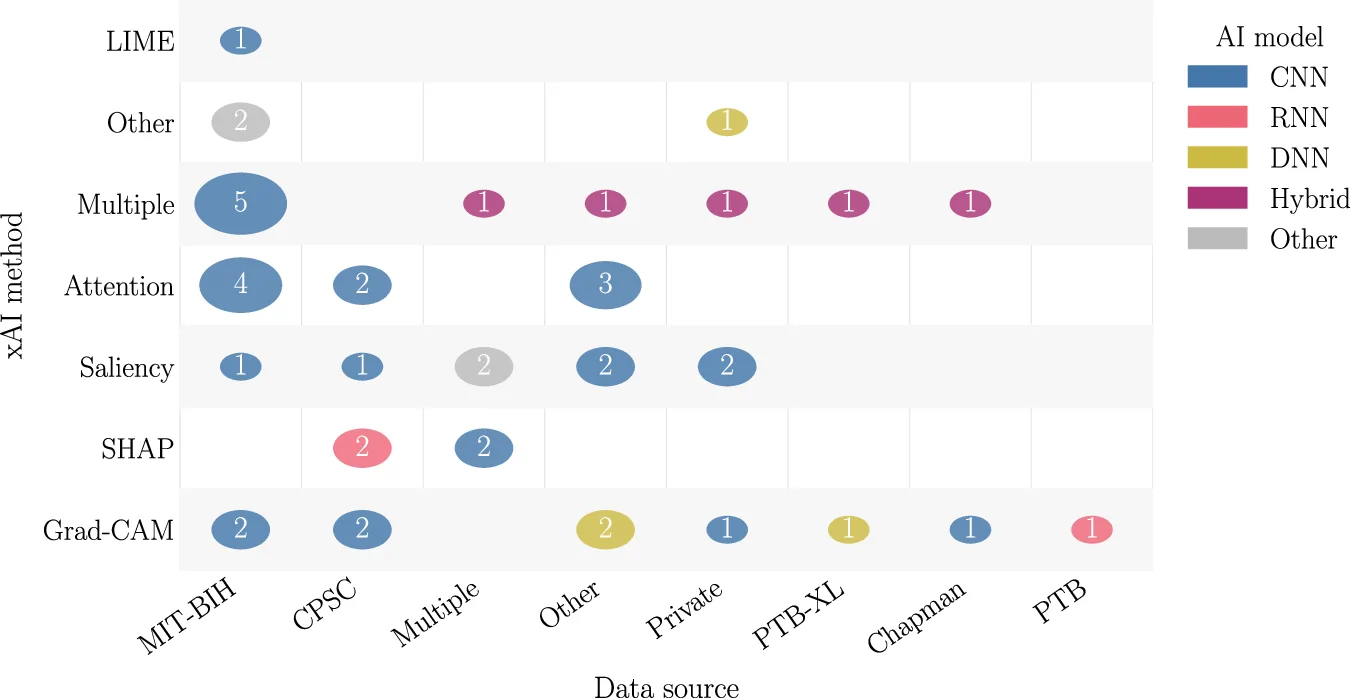
Co-occurrence matrix of data source and xAI method across included xAI studies (*n* = 30). The bubble size represents the number of study entries per cell, and colour indicates the dominant AI model. Note that smaller bubbles may be obscured by larger ones in cells with multiple model types; the figure is intended to illustrate dominant combinations rather than exhaustive model-level detail. CNN, Convolutional Neural Network; DNN, Deep Neural Network; Hybrid, studies combining two or more model architectures; RNN, Recurrent Neural Network.

Table 2 contains findings from a selection of the 30 xAI-related studies (2021–2025), indicating that xAI applied to life-threatening arrhythmias is an emerging field. Most studies (*n* = 20) employ a Convolutional Neural Network (CNN) as the foundation for their AI models (see Fig. 5). The most popular hybrid architecture is a CNN combined with a Long Short-Term Memory (LSTM) (*n* = 4). Three studies use multiple models, comparing a CNN with an LSTM ^21,22^, or a CNN with a k-Nearest Neighbour (kNN)^23^.

**Table 2:** Selected explainable artificial intelligence studies addressing life-threatening arrhythmias

Study	Data source	Objective	AI model	AI performance	xAI method
Abousaber, El-Ghaish, and Abdallah^30^	Publicly available dataset: MIT-BIH arrhythmia database	Multiple heartbeat classification	VAE & CNN	Accuracy: 0.996, F1-score: 0.954	SHAP & Grad-CAM
Ahn et al. ^24^	Private database (1,889 cardiac arrest ECGs from lead II, with 12.2% VF and 1.7% pVT)	Predict shockable rhythms	CNN	Accuracy: 0.749, AUPRC: 0.870, AUROC: 0.867, NPV: 0.893, PPV: 0.664, sensitivity: 0.914, specificity: 0.610	Grad-CAM
Cao et al. ^25^	PTB diagnosis	MI detection and localisation	RNN	MI detection: Accuracy: 0.998, sensitivity: 0.994, specificity: 0.998; MI localisation: Accuracy: 0.998, sensitivity: 0.988, specificity: 0.998.	Grad-CAM
Ganeshkumar et al. ^28^	CPSC 2018	Arrhythmia classification	CNN	Accuracy: 0.962, F1-score: 0.967, Hamming loss: 0.037, precision: 0.986, sensitivity: 0.949	Grad-CAM
Huang et al. ^67^	PTB-XL, Georgia, Chapman & CPSC 2018, and CinC 2017	Multiple arrhythmia classification (STC, PVC, WPW, and AF)	CNN	F1- score: 0.776 (STC); 0.885 (PVC); 0.825 (WPW); 0.854 (AF)	SHAP
Jin et al. ^29^	MIT-BIH arrhythmia & CPSC 2018	Arrhythmia classification	CNN	MIT: Accuracy: 0.888, F1-macro: 0.810; CPSC: Accuracy: 0.601, F1-macro: 0.653	Attention mechanism weights
Kolk et al. ^60^	Private dataset (32,129 ICD ECGs from 2,942 patients (61.7 ± 13.9 years), with a mean follow-up of 43.9 ± 35.9 months)	VT & VF classification	VAE & RF	AUROC: 0.738	Saliency map
Lu et al. ^73^	Private dataset (2,315,782 7-s to 10-s-long ECGs from 12 leads)	Multiple arrhythmia classification	DNN	AUROC: 0.998, F1-score: 0.948	Feature importance through isolation strategy (heatmaps)
Mamun et al. ^71^	Private dataset (29 paediatric and 10 adult patients with congenital heart disease, totalling 113,924 labelled beats across 20 arrhythmia categories)	Multiple arrhythmia classification (AVNRT, AVRT, AFL)	CNN with a transformer block	F1- score: 0.912, precision: 0.934,	Attention heatmap and SHAP
Nankani et al. ^26^	MIT-BIH arrhythmia, Malignant ventricular arrhythmia, Creighton University Ventricular Tachyarrhythmia, Ideo ventricular arrhythmia; private database.	VT & VF classification	RNN	Accuracy: 0.981, sensitivity: 0.962, specificity: 0.986	Gradient-based guided backpropagation

Studies are selected to represent the full range of AI models, xAI methods, datasets, and clinical objectives. We only included the specifics of private datasets for a compact overview, since public datasets are better known. See Table S4 in Supplementary Information C for all 30 studies with complete information.

*AF* Atrial Fibrillation, *AFL* Atrial Flutter; *AUPRC* Area Under the Precision-Recall Curve, *AUROC* Area Under the Receiver Operating Characteristic Curve, *AVNRT* Atrioventricular Nodal Reentrant Tachycardia, *AVRT* Atrioventricular Reentrant Tachycardia, *CNN* Convolutional Neural Network, *DNN*, Deep Neural Network, *ECG* Electrocardiogram, *Grad-CAM* Gradient-weighted Class Activation Mapping, *ICD* Implantable Cardiac Defibrillator, *LSTM* Long Short-Term Memory, *MI* Myocardial Infarction, *NPV* Negative Predictive Value, *PPV* Positive Predictive Value, *PVC* Premature Ventricular Contraction, *pVT* Polymorphic Ventricular Tachycardia, *RF* Random Forest, *RNN*, Recurrent Neural Network, *SHAP* Shapley Additive Explanations, *STC* ST-Changes, *VAE* Variational Autoencoder, *VF* Ventricular Fibrillation, *VT* Ventricular Tachycardia, *WPW* Wolff-Parkinson-White Syndrome.

All but seven studies use publicly available datasets for training or testing. The most commonly used public dataset is the MIT-BIH arrhythmia database (*n* = 14), followed by the CPSC-2018 (*n* = 8) (see Fig. 5). Studies focus mainly on multiple-arrhythmia classification (*n* = 13) or multiple-heartbeat classification (*n* = 11). The remaining six studies focused on predicting shockable rhythms^24^, detecting and localising myocardial infarctions^25^, classifying ventricular tachycardia and fibrillation^26^], and Brugada syndrome classification^27^.

The use of various performance metrics makes it challenging to compare the studies. All studies that use the CPSC-2018 dataset report performance using at least the F1-score. Ganeshkumar et al. ^28^ performs the best with an F1-score of 0.967. The lowest F1-score was 0.653^29^; however, Ganeshkumar et al. ^28^ employs only 3 of the 12 available leads for analysis. For the MIT-BIH arrhythmia database, the most commonly used performance metric is accuracy, for which Abousaber, El-Ghaish, and Abdallah^30^ achieved the best result (0.996), while Jin et al. ^29^ performs at the lower end (0.888).

Gradient-weighted Class Activation Mapping (Grad-CAM) is the most widely used xAI technique (*n* = 9), followed by Shapley Additive Explanations (SHAP) (*n* = 6). Other explainability methods identified in the review include Local Interpretable Model-Agnostic Explanations (LIME) and attention mechanisms (see Fig. 5).

### Cardiac electrophysiological modelling

**Fig. 6:**
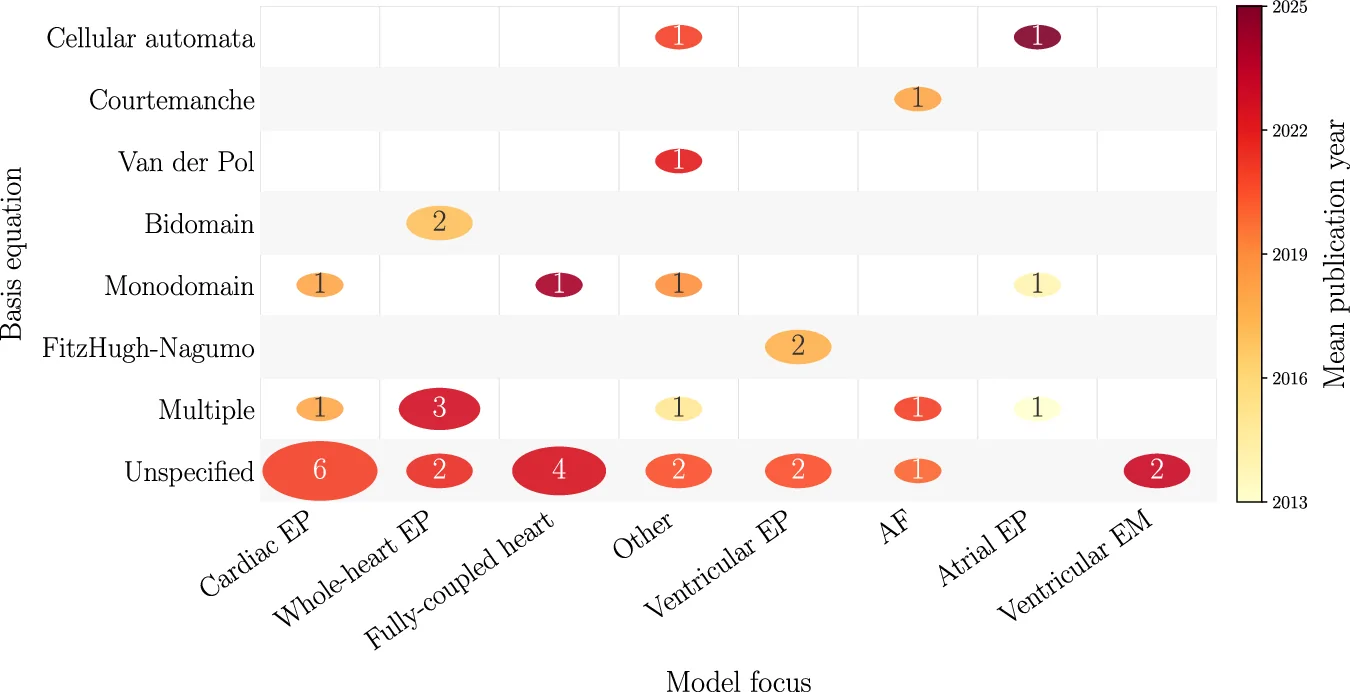
Co-occurrence matrix of model focus and basis equation across included cardiac electrophysiological modelling studies (*n* = 38). The bubble size represents the number of studies per cell, and colour indicates the mean publication year, ranging from 2013 (light yellow) to 2025 (dark red). AF, Atrial Fibrillation; EP, Electrophysiology; EM, Electromechanics.

Table 3 presents the 38 studies on cardiac electrophysiological modelling that met our inclusion criteria. Models primarily address cardiac (*n* = 8), whole-heart (*n* = 7), fully-coupled heart (*n* = 5), ventricular (*n* = 4), and atrial (*n* = 3) electrophysiology (see Fig. 6). The model focus “cardiac electrophysiology” primarily involves ion dynamics, membrane models, or ECG-related modelling. Other foci are state estimation, ventricular and cardiac tissue electromechanics, and myocardial growth modelling. Researched pathologies include atrial fibrillation (*n* = 3), Atrioventricular (AV) Nodal Reentrant Tachycardia (AVNRT) (*n* = 1), and fibrosis (*n* = 1).

**Table 3:** Selected cardiac electrophysiological models

Study	Model focus	Model novelty
Ai et al. ^31^	Whole-heart electrophysiology	Heart model constructed from discrete hybrid automaton models
Bucelli et al. ^39^	Fully-coupled heart	Accounts for the major feedback effects, including electro-mechanical and mechano-electrical feedback as well as force-strain and force-velocity relationships
Fedele et al. ^40^	Fully-coupled heart	Inclusion of atrial contraction
Jæger et al. ^44^	Whole-heart electrophysiology	Detailed analysis of the biophysical process going on in functionally important spaces very close to individual myocytes
Labarthe et al. ^34^	Atrial electrophysiology	Fully capturing propagation patterns seen in vivo
Moss et al. ^35^	Fully-coupled heart	Analysis of the heart’s contraction and its influences on the ECG
Romitti et al. ^33^	Atrial electrophysiology	Replicate atrial electrophysiology at different AF stages, including persistent AF, with a phenomenological model instead of biophysical models
Salvador et al. ^36^	Ventricular electromechanics	Synthesise both electrical and mechanical activities into a cohesive framework
Tenderini et al. ^37^	Cardiac electrophysiology	PDE-aware deep learning model for the numerical solution to the inverse problem of electrocardiography
Zappon et al. ^41^	Fully-coupled heart	Inclusion of the torso domain deformation induced by the myocardial displacement

See Table S5 in Supplementary Information C for all 38 studies with complete information.

*AF* Atrial fibrillation, *AV* Atrioventricular, *ECG* Electrocardiogram, *NSR* Normal sinus rhythm, *PAC* Premature atrial contractions, *PDE* Partial differential equation, *PVC* Premature ventricular contractions.

The monodomain (*n* = 5) and bidomain (*n* = 5) equations, which mathematically describe electrical propagation through cardiac tissue, are the most used models to describe the heart’s electrical activity (see Fig. 6). FitzHugh-Nagumo (*n* = 5) and Van der Pol (*n* = 5) equations are frequently combined (*n* = 3) for describing rhythmic phenomena and excitable systems, respectively.

Novel contributions include cellular automata, which enable computationally feasible cardiac dynamics simulations^31–33^ and advanced patient-specific modelling^34–38^. A further focus lies on coupled electromechanical systems^35,39–41^, providing a more comprehensive understanding of the electrical and mechanical interplay of the cardiac system.

Advanced mathematical techniques^42–47^ also represent a novelty in this field. A novel model is the Extracellular-Membrane-Intracellular (EMI) model, proposed by Jæger et al. ^44^, in which the extracellular, membrane, and intracellular spaces are not averaged as in the bidomain model but are represented as individual cells. The individual cells enable the analysis of individual myocytes despite the increased computational cost^44^.

### Risk of bias and quality of reporting

The PROBAST assessment identifies 63 studies as having a low risk of bias and 66 as having no applicability concerns (see Table S6 and Figure S1 in the Supplementary Information). Six studies have an unknown risk of bias (two on incidence, two on xAI, and two on cardiac electrophysiological models). Five have unknown concerns about applicability (three regarding incidence, two regarding the cardiac electrophysiological model). Fifteen studies are at high risk of bias (two on incidence and thirteen on cardiac electrophysiological model), and thirteen studies have high applicability concerns (one on incidence and twelve on cardiac electrophysiological model).

## Discussion

This discussion synthesises findings across three research domains: SrSCD/SrSCA incidence, xAI applied to ECGs for life-threatening arrhythmias, and cardiac electrophysiological models. We identify 84 studies; 16 pertain to SrSCD and SrSCA incidence, 30 address xAI in life-threatening arrhythmias, and 38 focus on cardiac electrophysiological models. We first examine the domains independently, then explore opportunities to integrate them to advance prevention of SrSCD/SrSCA in athletes.

Of the 16 studies on SrSCD, SrSCA, or SCD/SCA in athletes, 13 report incidence rates. Comparing incidence rates is challenging due to distinct populations (persons, participants, or athletes) and unclear denominators (i.e., the total number of persons/participants/athletes by which sudden cardiac death or arrest cases are divided). The highest reported SrSCD and SrSCA incidence among athletes is 1.57^12^ (50% during exercise) and 1.97^11^ (78% during or within one hour of exercise) per 100,000 athletes per year, respectively. Age emerges as a significant risk factor, whilst males account for the vast majority of deaths or arrests, representing 83^12^ – 100% ^17,48^ of cases across studies (excluding single-sex studies). While the upper end of this range may reflect sport-specific participation demographics, the male predominance in SrSCD and SrSCA is consistent across studies.

Data collection sources vary significantly. The Nordic countries of Denmark^19^, Sweden^7^, and Norway^49^ utilise nationwide registries, whilst others rely on voluntary self-reporting, which is likely to lead to underreporting. Fig. 3 shows that SrSCA incidences are lower than SrSCD incidences, suggesting either cardiac death overreporting or cardiac arrest underreporting since all SrSCD are also SrSCA. The data collection source also affects the reporting of pathological findings. Because not all countries mandate pathologist determination (e.g., Germany^13^), some studies rely on media monitoring ^18,50^ or insurance claims^12^, which can compromise the accuracy of the reports.

Figure 4 summarises the pathological findings of those who suffered an SrSCD/SrSCA or SCD/SCA. Sudden Arrhythmic Death Syndrome (SADS), a death characterised by abnormal electrical activity with a structurally normal heart, is primarily diagnosed in the younger end of the athlete’s spectrum ( ≥ 10 years). In comparison, Coronary Artery Disease (CAD) is the main pathological finding in older athletes ≥35 years ^51,52^. Wisten et al. ^53^ concludes that in Sweden, the average ages for SCD due to SADS and CAD are 22.3 and 31.3 years, respectively. These findings indicate that the pathological causes for SrSCD and SrSCA are age-dependent.

Pathological findings also differ by competitive level, with available data varying across regions depending on their sporting structures. Among recreational athletes from Europe (see Table 1), the main causes of SrSCD or SrSCA are SADS and CAD. In contrast, in the United States (see Table 1), the primary SCD and SCA causes in college athletes are Hypertrophic Cardiomyopathy (HCM), Idiopathic Left Ventricular Hypertrophy (ILVH), and Sudden Unexplained Death (SUD), which refers to an unexplained death that could be of arrhythmic or non-arrhythmic origin. The findings suggest that pathologies may be population-specific (related to training intensity or the athlete’s ethnic background) or sport-specific (American football in the United States versus football in Europe). The lack of representation from Africa, Central and South America, Eastern Europe, or Asia (except for Japan) limits the generalisability of the findings.

Although most of the studies are from Caucasian-dominant countries (see Fig. 2), the origin of the SrSCD/SrSCA study is not coequal with the ethnicity of those who suffered a SrSCD/SrSCA. Of the 16 incidence studies, only 6 report on ethnicity. In Drezner et al. ^50^ from the USA, for example, 51% are white non-Hispanic/Latino, 30% black/African American, and 11% white Hispanic/Latino, 3% Asian, 1% Native American, and the rest unknown. McClean et al. ^54^ states that paediatric black athletes have significantly more training-related and training-unrelated ECG changes than Caucasian athletes, which is also reflected in the international criteria for electrocardiographic interpretation in athletes^55^. This is further illustrated by Roberts and Stovitz^48^, who acknowledge that Minnesota has a lower proportion of African Americans than the general US population (5.4% vs 13.1%), underscoring that incidence rates cannot be extrapolated across regions with different ethnic compositions, as ethnicity is itself a modifier of cardiac risk.

One study finds an association between the underlying pathology and the timing of death. Wisten et al. ^53^ conclude that deaths during exercise were more likely to involve structural abnormalities (17%) than structurally normal hearts (8% SADS). SrSCA are reported more often during than after exercise. In Bohm et al. ^18^, SrSCA occurs in 82% of cases during sports activity and in 16% within 1 hour after cessation of sports (2% were unclear regarding the timing). In Peterson et al. ^11^ 74% happen during exercise and 4.2% within 1 h after exercise. These findings suggest that sport acts as a trigger with a post-exercise risk window and that more athletes with SrSCD and SrSCA have underlying structural abnormalities.

There are inconsistencies between studies in the definition of cardiac events. Most studies do not adjudicate a commotio cordis as an SCD or SCA, arguing that the death due to a blow is not an inherent cardiac problem (exceptions are Peterson et al. ^11^. and Petek et al. ^12^). Some studies also include any cardiac death or arrest that occurred up to 24 h after exercise cessation, as is the case in Malhotra et al. ^56^. A uniform definition for SrSCD and SrSCA is therefore required.

Table 2 summarises the data extraction for xAI addressing ECGs of life-threatening arrhythmias. Figure 5 reveals a predominance of CNNs across studies, suggesting limited architectural diversity. Grad-CAM, a well-established but inherently local explainability method, dominates the xAI landscape, limiting the ability to draw global conclusions. Studies utilise different combinations of performance metrics to measure the neural network’s classification or prediction ability. However, many studies use performance metrics that can be misleading when disease prevalence differs between datasets. Some studies do not account for this data imbalance at all ^25,26,57–61^. In contrast, others rely on only a single metric, such as the F1-score ^21,27,29,30,62–72^ rather than reporting multiple complementary metrics. Class underrepresentation can bias a model toward majority classes, leading to poor model generalisability and poor sensitivity and precision for minority classes. Evaluation metrics such as AUPRC and F1-score that account for data imbalance are necessary.

All but seven studies ^24,26,60,71,73,74,]^ rely only on publicly available ad-hoc datasets. These datasets are not explicitly created for a specific pathology or disease (e.g., SrSCD) but rather comprise a mixture of ECGs collected at a hospital’s emergency department. Publicly available ad-hoc datasets have contributed to the development of new AI methods for arrhythmia classification from ECGs. However, these datasets may be limited in sample size (e.g., PTB-diagnostics, *n* = 448 or CPSC-2018, *n* = 6,877). A neural network trained solely on a small dataset is prone to overfitting, leading to overly optimistic model performance^15^. The generalisability of small datasets is limited. Larger datasets (e.g., PhysioNet Challenge 21^75^, *n* = 88,253, which is open-source) can increase generalisability. Similarly, testing a model on patients different from those used to develop it provides a more realistic assessment of the model’s generalisability, i.e., how well it will perform in clinical practice. Using separate training and testing datasets is particularly vital when only a small number of cases are available for model development.

It is crucial to recognise the connection between the dataset and its corresponding population. Models trained on datasets that do not accurately represent the target population may learn spurious patterns or noise rather than clinically meaningful features^15^, compromising their robustness in real-world applications. This dataset-population mismatch is particularly problematic in 22 of 30 studies that attempt multiple classifications of heartbeats or arrhythmias without specific clinical objectives. We contend that pursuing a single “universal” arrhythmia detection model is unlikely to succeed. Instead, we propose that clinical AI development should focus on specialised models tailored to specific arrhythmias or clinical decision points, with each model rigorously optimised and validated for its particular diagnostic context. Examples of specific models for SrSCD/SrSCA prediction include classification of Ventricular Tachycardia (VT), Ventricular Fibrillation (VF) ^26,59,60^, and Brugada syndrome^27^.

Novel contributions regarding cardiac electrophysiology include simplified models such as cellular automata ^31,32^, patient-specific modelling ^34–37^, advanced mathematical techniques ^42–45^, and coupled electromechanical systems ^35,39–41^. Simplified models, such as hybrid automata, offer computational efficiency and real-time applicability but often trade off physiological accuracy ^31,32^. This trade-off is particularly crucial when considering the application of these models in research and clinical settings.

Patient-specific models ^36,38,76^ leverage Magnetic resonance imaging^36^ and electro-anatomical mappings^76^ to tailor simulations to individual geometries and electrophysiological properties, providing valuable insights for personalised therapy. However, these insights are not generally applicable and are computationally expensive. Advanced mathematical techniques, such as fractional differential operators ^42,43^ and machine learning^37^ have enhanced model fidelity and computational performance.

Challenges persist despite these advancements in cardiac electrophysiological models. High computational costs remain a barrier for detailed multi-scale models ^44,77^, and experimental validation is often limited to specific pathological conditions ^43,76,78,79^. The studies in Table 3 exhibit a disproportionate focus on a particular heart region (e.g., left ventricular ^36,80^ or atrial^39^ dynamics), neglecting the complex interplay of the entire cardiac system. Models incorporating high spatial resolution or detailed electromechanical coupling are computationally demanding and not feasible for real-time clinical application.

Figure 6 reveals how the model focus in cardiac electrophysiology has shifted towards whole-heart and fully-coupled approaches in recent years. The shift toward more integrative modelling approaches reflects a growing recognition of the importance of capturing the interplay across the entire cardiac system, as well as increasing computing power. A temporal trend analysis of xAI methods would be less informative, since the earliest included studies date back to 2021.

Having reviewed each domain independently, we now consider integrating them to prevent SrSCD/SrSCA. As outlined previously, the key challenge for young and adolescent athletes is distinguishing pathological changes from normal athletic and growth-related adaptations (see Supplementary Information A). Ethnicity and age are biological constants present in every dataset, every patient-specific model, and every patient — regardless of sport, competitive level, or exercise context.

Current xAI studies focus on general populations but could be adapted for athlete-specific risk stratification. Fostering ethnically diverse datasets would be advantageous for more accurate risk stratification in athletes, as paediatric black athletes, for example, show significantly more training-related and training-unrelated ECG changes than Caucasian athletes^54^. Not only would classification performance improve, but the explainability component would also be critical for quantifying the morphological features indicative of ECG classification using neural networks. The explainable features identified by these models could then focus on ethnic-specific morphological differences that distinguish pathological remodelling from physiological adaptation within the athlete population. Ethnicity could similarly influence the simulation of ECG signals ^41,46,81^ by, for example, informing the depth of T-wave inversion (higher risk when depth ≥ 2 mm^54^) or heart rate dynamics^82^.

Studies indicate that the pathological causes for SrSCD and SrSCA are equally age-dependent. CAD-rich datasets would be the smarter choice for predicting SrSCD in older European athletes ≥ 35 years, whereas datasets for HCM or ILVH diagnosis would be the better choice for US college athletes. A neural network focused on abnormal electrical activity, such as Brugada syndrome^27^ or Wolff-Parkinson-White Syndrome^67^, for classification purposes could be beneficial for younger athletes with structurally normal hearts who are at risk of SADS. Current xAI approaches treat arrhythmia detection as a general classification problem divorced from pathology-specific risk factors. Developing athlete-specific databases that include both healthy and pathological ECG data is crucial to addressing current challenges in screening sensitivity and specificity.

Age could be considered when computing an ECG and body surface potential maps using a realistic heart-torso model to account for the effects of heart position relative to the torso^38^. Bjerring et al. ^83^. conducted a longitudinal study of cross-country skiers assessed with echocardiography at ages 12, 15, and 18. The skiers underwent eccentric remodelling between ages 12 and 15 and concentric remodelling between ages 15 and 18. Sex-related differences in an athlete’s heart were evident from age 12, where males showed remodelling beyond reference values^84^. Patient-specific modelling approaches that currently utilise imaging and clinical data could incorporate sex- and age-dependent cardiac remodelling trajectories. These simulations could identify critical thresholds where adaptive cardiac remodelling becomes maladaptive and pro-arrhythmic. Cardiac electrophysiological modelling can further address differences in cardiac adaptations between recreational and elite athletes, as well as the distinct structural pathologies observed in European recreational athletes versus American collegiate athletes.

Over-reliance on a small number of public ECG datasets limits generalisability. Cardiac electrophysiological modelling could assist in generating sports-related synthetic ECGs and body-surface potential maps. These synthetic datasets could then be used to train and validate xAI models, thereby expanding generalisability to adolescents and young adult athletes. XAI also benefits from cardiac electrophysiological models within physics-informed approaches such as Physics-Informed Neural Networks (PINNs). PINNs are an AI framework for solving complex problems governed by physical laws^85^. Embedding physical laws directly into the learning process would enhance both the interpretability and explainability of xAI models and enrich the limited architectural diversity of CNNs.

A general remark is that many studies, particularly in xAI and cardiac modelling, have failed to report limitations that compromise transparency and proper interpretation of findings^86^. Without limitations, readers cannot correctly position research findings within their context to determine the credibility of a study’s conclusion or generalise results properly^86^.

This systematic review faces certain limitations. The term “life-threatening arrhythmias” is inherently ambiguous. Although we provide a definition, different interpretations in the literature may affect study inclusion. Similarly, “sport” and “athlete” are broadly defined terms, and despite using inclusive search terms (“Sport*” OR “Athlet*” OR “Exercis*”), we may have missed studies on cardiac death or cardiac arrest in specific sports that were not explicitly labelled with these terms.

The lack of uniform definitions of explainability in xAI makes it challenging to delineate boundaries, potentially leading to inconsistent inclusion of studies. AI’s frequent neologisms and evolving terminology increase the risk of overlooking relevant studies that use alternative terms. This linguistic variability could inadvertently introduce bias in the reviewed literature and affect the comprehensiveness of the findings.

Cardiac electrophysiological models pose additional challenges, as many are extensions of existing frameworks, making the assessment of novelty complex and subjective. This ambiguity can lead to the exclusion of relevant studies or the inclusion of non-novel studies.

We constrain our search strategy to English-language publications, specific databases, and grey literature. Studies published in languages other than English or in regional databases may have been overlooked, contributing to a geographical imbalance, as most studies originate from Europe and North America, thereby limiting generalisability.

PROBAST, whilst suitable for prediction studies, may be unsuitable for assessing SrSCD/SrSCA incidence and cardiac electrophysiological modelling studies, potentially affecting our bias assessments.

Concluding, the review’s findings confirm that age is a significant risk factor for sports-related sudden cardiac events, even in younger populations, whilst male sex is a risk factor for SrSCD/SrSCA. Ethnicity is an underreported risk factor. SrSCD and SrSCA incidences require greater standardisation to enable reliable data collection and comparison, such as the International Criteria for Reporting Study Quality for Sudden Cardiac Arrest/Death tool^87^.

xAI and cardiac electrophysiological models can be powerful diagnostic tools for uncovering disease mechanisms and risk factors, enabling better prevention and personalised treatments. However, their application to SrSCD/SrSCA remains limited, and the lack of integration among epidemiologically identified risk factors, xAI approaches, and mechanistic modelling in the athletic population represents a missed opportunity. Including risk factors (e.g., ethnicity or age) in both domains could enable athlete-specific approaches. Cardiac electrophysiological models could generate synthetic, athlete-specific ECGs for xAI training and testing, with physics-informed approaches such as PINNs enhancing interpretability and explainability.

Focusing AI development solely on ECG classification performance without advancing understanding of cardiovascular disease overlooks opportunities to address the complex mechanisms underlying SrSCA. Nonetheless, we believe the significance of explainability in AI applied healthcare is rather instrumental than intrinsic^88^, serving primarily as a valuable analysis tool for developers, auditors, and regulators of these systems^89^. The argument follows the reality that AI in healthcare is alluring precisely because it “learns” and generalises data at scales and speeds beyond the reach of human cognition. The ability to identify patterns at such a scale and speed is where AI’s value lies. Hence, “limiting machines in reasoning as humans do, we may rob them—and ourselves—of their unique potential to solve problems which we cannot”^88^.

Future research should prioritise: (1) standardised definitions and reporting protocols for SrSCD/SrSCA, including ethnicity to enable subgroup analyses; (2) purpose-built, ethnicity- and age-informed datasets for training xAI models; and (3) integrating pathology-specific risk factors into both xAI and cardiac modelling frameworks to enable athlete-specific risk stratification.

## Methods

We systematically reviewed the incidence of SrSCD and SrSCA in adolescent and young adult athletes, as well as the application of xAI in life-threatening arrhythmias and cardiac electrophysiological models, following the PRISMA 2020 guidelines^90^. The study protocol was registered in the International Prospective Register of Systematic Reviews (PROSPERO; CRD42024565960). We developed the following Population, Intervention, Comparator, and Outcome (PICO) statement for the literature search strategy:

- Population: We included human studies in which at least one of the study groups comprises individuals participating in either recreational or professional sports. We limited the reviewed population to adolescents and young adults, i.e., those between 10 and 35 years who suffered a SrSCD or SrSCA. We adopted the definition of SrSCD/SrSCA as a non-traumatic death/arrest presumably of cardiac origin during or within one hour following exercise.
- Intervention: The review focused on the incidence of SrSCD and SrSCA in adolescents and young adults (10–35 years), mathematical and general cardiac electrophysiology models, and xAI applied to ECGs of life-threatening arrhythmias. We defined a life-threatening arrhythmia as an arrhythmia that, if not treated or disappears by itself (i.e., non-sustained), leads to cardiac arrest within minutes or hours of onset. The dataset had to contain at least one life-threatening arrhythmia (e.g., ventricular tachycardia or fibrillation).
- Comparator: We made no direct comparisons. This review aimed to map the current landscape across three research domains: SrSCD/SrSCA in adolescent and young adult athletes, xAI applications on ECGs with life-threatening arrhythmias, and cardiac electrophysiological modelling approaches.
- Outcome: We employed SrSCD/SrSCA incidences to summarise the current burden. We used the neural network’s classification or prediction performance metrics for life-threatening arrhythmia prediction as our primary outcome. The neural network had to emphasise a reasoning framework that helps to understand the classification or prediction decisions during or after operation (post-hoc). We extracted objectives and novelty from cardiac electrophysiological models.

### Literature search

The databases searched were ACM (Guide to Computing Literature), IEEE Xplore, MEDLINE, Scopus, Web of Science, EMBASE, SPORTDiscus, and Google Scholar for grey literature. Inclusion criteria included peer-reviewed primary-source publications in English published between 2013 and 2025. “Young adult”, “adolescent”, “incidence”, and “athletes” were the words applied for the SrSCD incidence search. In accordance with PRISMA guidelines, we documented the full search strings in Supplementary Information B. The search strategy for the xAI consisted of the terms “Artificial Intelligence”, “electrocardiography”, “arrhythmias”, and “sudden death”. In contrast, the mathematical models were searched for using the terms “mathematics”, “models”, and “cardiac electrophysiology”. Each domain was searched independently.

### Literature selection

Literature search results were managed using JabRef, an open-source, cross-platform citation and reference management tool. The screening process used Rayyan.ai, a cloud-based software application tailored for researchers conducting systematic literature reviews. Three authors (EV, NS, AG) conducted a two-phase screening process consisting of: a title/abstract review followed by a comprehensive full-text review. Disagreements were resolved by consensus or by input from a fourth reviewer (MV). Reference lists of key studies were reviewed for additional publications.

### Data extraction

We captured 12 data items: publication details (title, author, year, country), objective, input data type, dataset source, methodology, results (incidence or accuracy), xAI methods, and research challenges. Data were tabulated for comparison and pattern identification. Incidence studies were standardised to cases per 100,000 persons/participants/athletes per year where possible.

### Data synthesis

We conducted qualitative synthesis across three categories: SrSCD/SrSCA incidence, xAI applications, and cardiac electrophysiological models. Incidence studies were narratively summarised by age, sex, sport type, and region. xAI studies were compared by techniques, datasets, and performance metrics. Approaches, equations, validation methods, and clinical applications were analysed in electrophysiological models. We emphasised methodological limitations, gaps in the literature, and implications for future research.

### Quality of evidence and risk of bias

We assessed the risk of bias to ensure robust and reliable findings using the Prediction model Risk Of Bias ASsessment Tool (PROBAST). PROBAST evaluates bias across four domains: participants (whether the study population is representative), predictors (whether predictors are defined and measured consistently), outcome (whether outcomes are defined and measured without bias), and analysis (whether appropriate statistical methods are used). This tool consists of two analyses: risk of bias and applicability. The risk of bias addresses the impartiality of the selected studies. Applicability refers to the extent to which the chosen studies align with the PICO statement. Bias can arise when study populations are unrepresentative, predictors are inconsistently defined or measured, outcomes are unreliably ascertained, or inappropriate statistical methods are applied. Three reviewers (EV, NS, AG) independently assessed bias risk and reporting quality, with disagreements resolved through discussion or arbitration (MV). We used PROBAST findings to contextualise the individual study throughout this review.

## Supplementary information

Supplementary information

## Data Availability

No datasets were generated or analysed during the current study.
