# Supplementary material for: A systematic review of explainable artificial intelligence and cardiac electrophysiological models addressing sports-related sudden cardiac death and arrest in adolescents and young adults
Source: NPJ Digit Med. 2026 Jun 11;9:721. doi: 10.1038/s41746-026-02878-x (PMC13601518; doi:10.1038/s41746-026-02878-x)
Supplement: Supplementary file 1 [file 41746_2026_2878_MOESM1_ESM.pdf]

Supplementary Information

The authors have provided this Supplementary Information to give readers additional information about their work.

Contents

Supplementary Information A. Exercise-induced cardiac remodelling 3
Supplementary Information B. Full search strategy 4
B1. ACM (Guide to Computing Literature) 4
B2. IEEE Xplore 5
B3. MEDLINE 5
B4. Scopus 6
B5. Web of Science 7
B6. EMBASE 7
B7. SPORTDiscus 8
B8. Google Scholar search engine 8
Supplementary Information C. Tables 10
Table S1 Sport-related sudden cardiac death incidence for adolescents and young adults (10–35 years). 10
Table S2 Sport-related sudden cardiac arrest incidence for adolescents and young adults (10 – 35 years). 12
Table S3 Sudden cardiac death and arrest incidence for adolescents and young adults (10 – 35 years). 15
Table S4 Explainable artificial intelligence addressing life-threatening arrhythmias. 16
Table S5 Cardiac electrophysiological models. 25
Table S6 Prediction model Risk Of Bias ASsessment Tool (PROBAST) for the 89 papers meeting the inclusion criteria. 34
Supplementary Information D. Figures 45
Supplementary Information E. PRISMA Checklists 46
References 50

A systematic review of explainable artificial intelligence and cardiac electrophysiological models addressing sports-related sudden cardiac death and arrest in adolescents and young adults

Erik Vanegas Müller[ab]*, Nicharee Srikijkasemwat[b], Andrew Gan[c], Betty Raman[d], Mirae Harford[bef], Liang He[ab], Abhirup Banerjee[bd], Katja Gehmlich[cd], Paul Leeson[d] and Mauricio Villarroel[ab]*

[a]The Podium Institute for Sports Medicine and Technology, Department of Engineering Science, University of Oxford, Oxford, United Kingdom

[b]Institute of Biomedical Engineering, Department of Engineering Science, University of Oxford, Oxford, United Kingdom

[c]Department of Cardiovascular Sciences, University of Birmingham, Birmingham, United Kingdom

[d]Division of Cardiovascular Medicine, Radcliffe Department of Medicine, University of Oxford, Oxford, United Kingdom

[e]Critical Care Research Group, Nuffield Department of Clinical Neurosciences, University of Oxford, Oxford, United Kingdom

[f]NIHR Oxford Biomedical Research Centre, University of Oxford, Oxford, United Kingdom

Correspondence email: erik.vanegasmuller@eng.ox.ac.uk; mauricio.villarroel@eng.ox.ac.uk

Funding information EV, LH, and MV are funded by The Podium Institute of Sports Medicine and Technology. AB is supported by the Royal Society University Research Fellowship (Grant No. URFR1‘314). PL acknowledges funding from the Oxford NIHR Biomedical Research Centre, Medical Research Council and British Heart Foundation. MH is funded by the National Institute for Health and Care Research (NIHR) Oxford Biomedical Research Centre (BRC). The views expressed are those of the authors and not necessarily those of the NIHR or the Department of Health and Social Care. Research in KG’s laboratory is funded by the British Heart Foundation (IA/F/23/275037).

Supplementary Information A. Exercise-induced cardiac remodelling

Exercise-induced cardiac remodelling, also known as the "athlete's heart", adaptively increases the cardiac chamber size and wall thickness triggered by the volumes and pressure loads of exercise [1]. Myocardial hypertrophy for increased contraction and increased chamber volume to accommodate a higher blood volume are examples of structural remodelling [2]. Higher ejection fraction to sustain the higher oxygen demand during exertion, and increased vagal tone and sympathetic stimulation while resting and exercising are functional and electrical induced adaptations, respectively [3].

McClean et al. [4] analysed structural and electrical adaptations of the paediatric athlete's heart. The systematic review encompasses data from 14,278 athletes (13.8 ± 1.3 years) and 1,668 non-athletes (12.6 ± 0.6 years) from 43 qualitative and 40 quantitative studies published between 1982 and 2016. Football (33%) was the most predominant sport among the 30 different disciplines in which athletes competed. Males (83%, based on reported ages) were predominant, and Caucasian (92%, based on reported ethnicities) was the predominant ethnicity in both cohorts. In the review, a competitive athlete is defined as "one who participates in an organised team or individual sport that requires regular competition against others as a central component, places a high premium on excellence and achievement, and requires some form of systematic (and usually intense) training" [4]. Through the meta-analysis, McClean et al. [4] inferred that paediatric athletes were up to 13 times more likely to present a deep-T-wave inversion (≥ 2 mm) on an ECG than age-matched non-athletes. For the age group ≥ 14 years, paediatric athletes were up to 16 times more likely to exhibit T-wave inversion on the inferolateral leads (leads II – aVF/V5 – V6/I – aVL) than athletes ≤ 14 years. According to [4] inferolateral T-wave inversion should prompt further investigation and be treated as pathological until proven otherwise. Furthermore, paediatric athletes had a more pronounced left ventricular mass (e.g., a mean increase of 27.6%) compared to the age-matched non-athlete cohort.

The sport's inherent static and dynamic components impact the exercise-induced cardiac remodelling. The main factors relate to the exercise intensity, duration, and frequency. In other words, haemodynamic stress exposure (time x intensity of sport) and frequency define the athlete's heart [1,5]. Or, as La Gerche et al. [1] expresses it: "the heart cannot see what shoes you are wearing, but it can feel the effects", underscoring that cardiac adaptations respond to physiological demands rather than specific sports categories. The challenge for clinicians lies not in establishing whether a sports-arrhythmia relationship exists but in determining how significant this relationship is in each individual [6]. This clinical uncertainty is particularly problematic, as exercise-induced cardiac adaptations are not always unambiguous, making athletes a population that would benefit from new, robust markers of life-threatening arrhythmia risk. Such risk markers would aid in distinguishing between exercise-induced physiological adaptations and those that mimic diseased hearts and could predispose individuals to arrhythmia, syncope, and SCD.

Understanding the anatomical and physiological characteristics of the adolescent and young adult (10–35 years) athlete's heart compared to those of age-matched non-athletes is crucial for distinguishing between pathological and non-pathological athlete's hearts. To address this, we propose using xAI combined with cardiac electrophysiological models to gain a deeper understanding of the nature of exercise-induced cardiac remodelling. Current screening approaches rely heavily on ECGs as a first-line investigation, which assess only electrical activity rather than mechanical insights. As demonstrated in McClean et al. [4] by the varying ECG morphologies in athletes, differentiating between pathological and physiological changes remains challenging. The International Criteria for Electrocardiographic Interpretation in Athletes [7] attempt to standardise screening, but these criteria alone remain insufficient for accurate risk stratification.

Supplementary Information B. Full search strategy

This supplementary information contains the detailed search strategies for the seven databases. We present the search strategy that yields the most results. For ACM, for example, the asterisk (*) returned fewer results; hence, we searched without the asterisk and added the words the asterisk was meant to complete.

B1. ACM (Guide to Computing Literature)

ACM does not offer the option to search for abstracts, keywords, and titles simultaneously. Therefore, we searched for all.

Query Syntax: ”query”: AllField:((((”AI” OR ”Artificial Intelligence” OR ”ML” OR ”Machine Learning” OR ”DL” OR ”Deep Learning” OR ”NN” OR ”Neural Networks”) AND (”Interpret*” OR ”Explainab*” OR ”xAI”) AND (”Electrocardiograph*” OR ”ECG*” OR ”EKG*” OR ”Electrocardiogram*” OR ”Holter”)) AND (”Life-threatening” OR ”Lethal” OR ”Arrhythmia” OR ”Cardiac Arrest” OR ”Sudden Death” OR ”Ventricular” OR ”Asystole”))

OR

(((”Young Adult” OR ”Youth” OR ”Pubert*” OR ”Teenager”) AND (”Incidence” OR ”Prevalence”) AND (”Sport*” OR ”Athlet*” OR ”Exercis*”)) AND (”Life-threatening” OR ”Lethal” OR ”Arrhythmia” OR ”Cardiac Arrest” OR ”Sudden Death” OR ”Ventricular” OR ”Asystole”))

OR

(((”computational model*” OR ”mathematical model*” OR ”biological model*” OR ”physical model*” OR ”theoretical model*”

) AND (”Cardiac Electrophysiology” OR ”Heart Conduction System”)) AND (”Life-threatening” OR ”Lethal” OR ”Arrhythmia” OR ”Cardiac Arrest” OR ”Sudden Death” OR ”Ventricular” OR ”Asystole”)))

”filter”: E-Publication Date: (01/01/2013 TO 12/31/2025)

B2. IEEE Xplore

The IEEE search command allows only 25 search terms per search clause. Hence, we had to split the search into the following three individual searches:

(((”All Metadata”: ”AI” OR ”Artificial Intelligence” OR ”ML” OR ”Machine Learning” OR ”DL” OR ”Deep Learning”

OR ”NN” OR ”Neural Networks”) AND (”All Metadata”: ”Interpret*” OR ”Explainab*” OR ”xAI”) AND (”All Metadata”: ”Electrocardiograph*” OR ”ECG*” OR ”EKG*” OR ”Electrocardiogram*” OR ”Holter”)) AND (”All Metadata”: ”Life- threatening” OR ”Lethal” OR ”Arrhythmia” OR ”Cardiac Arrest” OR ”Sudden Death” OR ”Ventricular” OR ”Asystole”

))

(((”All Metadata”: ”Young Adult” OR ”Youth” OR ”Pubert*” OR ”Teenager”) AND (”All Metadata”: ”Incidence” OR ”Prevalence”) AND (”All Metadata”: ”Sport*” OR ”Athlet*” OR ”Exercis*”)) AND (”All Metadata”: ”Life-threatening” OR ”Lethal” OR ”Arrhythmia” OR ”Cardiac Arrest” OR ”Sudden Death” OR ”Ventricular” OR ”Asystole”))

(((”All Metadata”: ”computational model*” OR ”mathematical model*” OR ”biological model*” OR ”physical model*”

OR ”theoretical model*”) AND (”All Metadata”: ”Cardiac Electrophysiology” OR ”Heart Conduction System”)) AND

(”All Metadata”: ”Life-threatening” OR ”Lethal” OR ”Arrhythmia” OR ”Cardiac Arrest” OR ”Sudden Death” OR ”Ventricular” OR ”Asystole”))

B3. MEDLINE

(exp Artificial Intelligence/ or exp neural networks, computer/ or (”AI” or ”Artificial Intelligence” or ”ML” or ”Machine Learning” or ”DL” or ”Deep Learning” or ”NN” or ”Neural Networks”).ab,kf,ti.) and (”Interpret*” or ”Explainab*” or ”xAI”).ab,kf,ti. and (exp Electrocardiography/ or (”Electrocardiograph*” or ”ECG*” or ”EKG*” or ”Electrocardiogram*” or ”Holter”).ab,kf,ti.)

(exp adolescent/ or exp Young Adult/ or (”Young Adult” or ”Youth” or ”Pubert*” or ”Teenager”).ab,kf,ti.) and (exp Incidence/ or exp Prevalence/ or (”Incidence” or ”Prevalence”).ab,kf,ti.) and (exp Sports/ or exp Athletes/ or exp Exercise/ or (”Sport*” or ”Athlet*” or ”Exercis*”).ab,kf,ti.)

(models, theoretical/ or models, cardiovascular/ or (”computational model*” or ”mathematical model*” or ”biological model*” or ”physical model*” or ”theoretical model*”).ab,kf,ti.) and (exp heart conduction system/ or cardiac electrophysiology/ or (”Cardiac Electrophysiology” or ”Heart Conduction System”).af,kf,ti.)

exp arrhythmias, cardiac/ or exp heart arrest/ or (”Life-threatening” or ”Lethal” or ”Arrhythmia” or ”Cardiac Arrest” or ”Sudden Death” or ”Ventricular” or ”Asystole”).ab,kf,ti.

(1 and 4) or (2 and 4) or (3 and 4)

limit 5 to (english language and yr=”2013 - 2025”)

B4. Scopus

(TITLE-ABS-KEY (((”AI” OR ”Artificial Intelligence” OR ”ML” OR ”Machine Learning” OR ”DL” OR ”Deep Learning”

OR ”NN” OR ”Neural Networks”) AND (”Interpret*” OR ”Explainab*” OR ”xAI”) AND (”Electrocardiograph*” OR ”ECG*” OR ”EKG*” OR ”Electrocardiogram*” OR ”Holter”)) AND (”Life-threatening” OR ”Lethal” OR ”Arrhythmia” OR ”Cardiac Arrest” OR ”Sudden Death” OR ”Ventricular” OR ”Asystole”))

OR

TITLE-ABS-KEY (((”Young Adult” OR ”Youth” OR ”Pubert*” OR ”Teenager”) AND (”Incidence” OR ”Prevalence”)

AND (”Sport*” OR ”Athlet*” OR ”Exercis*”)) AND (”Life-threatening” OR ”Lethal” OR ”Arrhythmia” OR ”Cardiac Arrest” OR ”Sudden Death” OR ”Ventricular” OR ”Asystole”))

OR

TITLE-ABS-KEY (((”computational model*” OR ”mathematical model*” OR ”biological model*” OR ”physical model*” OR ”theoretical model*”) AND (”Cardiac Electrophysiology” OR ”Heart Conduction System”)) AND (”Life-threatening”

OR ”Lethal” OR ”Arrhythmia” OR ”Cardiac Arrest” OR ”Sudden Death” OR ”Ventricular” OR ”Asystole”))) AND PUBYEAR

¿ 2012 AdND PUBYEAR ¡ 2026 AND (LIMIT-TO (LANGUAGE , ”English”))

B5. Web of Science

((TS=(((”AI” OR ”Artificial Intelligence” OR ”ML” OR ”Machine Learning” OR ”DL” OR ”Deep Learning” OR ”NN” OR ”Neural Networks”) AND (”Interpret*” OR ”Explainab*” OR ”xAI”) AND (”Electrocardiograph*” OR ”ECG*” OR ”EKG*” OR ”Electrocardiogram*” OR ”Holter”)) AND (”Life-threatening” OR ”Lethal” OR ”Arrhythmia” OR ”Cardiac Arrest”

OR ”Sudden Death” OR ”Ventricular” OR ”Asystole”))) OR

TS=(((”Young Adult” OR ”Youth” OR ”Pubert*” OR ”Teenager”) AND (”Incidence” OR ”Prevalence”) AND (”Sport*” OR ”Athlet*” OR ”Exercis*”)) AND (”Life-threatening” OR ”Lethal” OR ”Arrhythmia” OR ”Cardiac Arrest” OR ”Sudden Death” OR ”Ventricular” OR ”Asystole”)))

OR

TS=(((”computational model*” OR ”mathematical model*” OR ”biological model*” OR ”physical model*” OR ”theoretical model*”) AND (”Cardiac Electrophysiology” OR ”Heart Conduction System”)) AND (”Life-threatening” OR ”Lethal” OR ”Arrhythmia” OR ”Cardiac Arrest” OR ”Sudden Death” OR ”Ventricular” OR ”Asystole”)) and Preprint Citation Index (Exclude – Database) and English (Languages)

Index date: 2013-01-01 to 2025-12-31

B6. EMBASE

(exp Artificial Intelligence/ or exp neural networks, computer/ or (”AI” or ”Artificial Intelligence” or ”ML” or ”Machine Learning” or ”DL” or ”Deep Learning” or ”NN” or ”Neural Networks”).ab,kf,ti.) and (”Interpret*” or ”Explainab*” or ”xAI”).ab,kf,ti. and (exp Electrocardiography/ or (”Electrocardiograph*” or ”ECG*” or ”EKG*” or ”Electrocardiogram*” or ”Holter”).ab,kf,ti.)

(exp adolescent/ or exp Young Adult/ or (”Young Adult” or ”Youth” or ”Pubert*” or ”Teenager”).ab,kf,ti.) and (exp Incidence/ or exp Prevalence/ or (”Incidence” or ”Prevalence”).ab,kf,ti.) and (exp Sports/ or exp Athletes/ or exp Exercise/ or (”Sport*” or ”Athlet*” or ”Exercis*”).ab,kf,ti.)

(models, theoretical/ or models, cardiovascular/ or (”computational model*” or ”mathematical model*” or ”biological model*” or ”physical model*” or ”theoretical model*”).ab,kf,ti.) and (exp heart conduction system/ or cardiac electrophysiology/ or (”Cardiac Electrophysiology” or ”Heart Conduction System”).af,kf,ti.)

exp arrhythmias, cardiac/ or exp heart arrest/ or (”Life-threatening” or ”Lethal” or ”Arrhythmia” or ”Cardiac Arrest” or ”Sudden Death” or ”Ventricular” or ”Asystole”).ab,kf,ti.

(1 and 4) or (2 and 4) or (3 and 4)

limit 5 to (english language and yr=”2013 - 2025”)

B7. SPORTDiscus

((”AI” OR ”Artificial Intelligence” OR ”ML” OR ”Machine Learning” OR ”DL” OR ”Deep Learning” OR ”NN” OR ”Neural Networks”) AND (”Interpret*” OR ”Explainab*” OR ”xAI”) AND (”Electrocardiograph*” OR ”ECG*” OR ”EKG*” OR ”Electrocardiogram*” OR ”Holter”)) AND (”Life-threatening” OR ”Lethal” OR ”Arrhythmia” OR ”Cardiac Arrest” OR ”Sudden Death” OR ”Ventricular” OR ”Asystole”)

OR

((”Young Adult” OR ”Youth” OR ”Pubert*” OR ”Teenager”) AND (”Incidence” OR ”Prevalence”) AND (”Sport*” OR ”Athlet*” OR ”Exercis*”)) AND (”Life-threatening” OR ”Lethal” OR ”Arrhythmia” OR ”Cardiac Arrest” OR ”Sudden Death” OR ”Ventricular” OR ”Asystole”)

OR

((”computational model*” OR ”mathematical model*” OR ”biological model*” OR ”physical model*” OR ”theoretical model*”

) AND (”Cardiac Electrophysiology” OR ”Heart Conduction System”)) AND (”Life-threatening” OR ”Lethal” OR ”Arrhythmia” OR ”Cardiac Arrest” OR ”Sudden Death” OR ”Ventricular” OR ”Asystole”)

Limit to English and to years 2013-2025.

B8. Google Scholar search engine

We divided the Google Scholar search into three parts, similar to the IEEE Xplore approach. The reason is that Google Scholar sorts its results by relevance, yielding over 45,000 results when all terms are searched simultaneously. Since screening 45,000 papers is disproportional, we would need to break the screening after, e.g, 500 entries. The risk is that papers relevant to, e.g., the incidence, may not appear in the first 500, since the Google Scholar algorithm considers them less appropriate than any paper containing something about xAI.

We stopped including papers from Google Scholar when the results were no longer related to our research. This meant we included 200 out of 17,300 results for xAI, 120 out of 16,400 results for Incidence, and 100 out of 4,530 results for modelling. The used search strategy is:

((”AI” OR ”Artificial Intelligence” OR ”ML” OR ”Machine Learning” OR ”DL” OR ”Deep Learning” OR ”NN” OR ”Neural Networks”) AND (”Interpret*” OR ”Explainab*” OR ”xAI”) AND (”Electrocardiograph*” OR ”ECG*” OR ”EKG*” OR ”Electrocardiogram*” OR ”Holter”)) AND (”Life-threatening” OR ”Lethal” OR ”Arrhythmia” OR ”Cardiac Arrest” OR ”Sudden Death” OR ”Ventricular” OR ”Asystole”)

(((”Young Adult” OR ”Youth” OR ”Pubert*” OR ”Teenager”) AND (”Incidence” OR ”Prevalence”) AND (”Sport*” OR ”Athlet*” OR ”Exercis*”)) AND (”Life-threatening” OR ”Lethal” OR ”Arrhythmia” OR ”Cardiac Arrest” OR ”Sudden Death” OR ”Ventricular” OR ”Asystole”))

(((”computational model*” OR ”mathematical model*” OR ”biological model*” OR ”physical model*” OR ”theoretical model*”) AND (”Cardiac Electrophysiology” OR ”Heart Conduction System”)) AND (”Life-threatening” OR ”Lethal” OR ”Arrhythmia” OR ”Cardiac Arrest” OR ”Sudden Death” OR ”Ventricular” OR ”Asystole

Supplementary Information C. Tables

Table S1 Sport-related sudden cardiac death incidence for adolescents and young adults (10–35 years).

Study; year	Country; years	Source	Population	Age; sex	Ethnicity	Sport	SrSCD incidence	Pathological findings
Boden et al.[8]; 2013	USA; 1990–2010	National Center for Catastrophic Sports Injury Research	Athletes (high school (n = 86) and college (n = 14))	—; 100 % male	—	American football	0.41 per 100,000 participants per year (*n* = 100)	HCM (n = 22), CAA (n = 11), Mitral valve abnormality (n = 2), Myocarditis (n = 2), undetermined (n = 63)
Ha et al. [9]; 2022	Australia; 2000–2016	National Coronial Information System in Australia	Athletes (Non-competitive and competitive/active (13%))	10 – 35 years; 92% male	53% Caucasian, 23% Unknown, 12% Other, 9% Aboriginal or Torres Strait Islander Peoples, <5% Asian	24% Australian rules football, 14% running/jogging and 14% football	(*n* = 110)	CAD (n = 41), SADS (n = 22), CM (n = 17), other (n = 30), 95% underwent autopsy
Marijon et al. [10]; 2013	France; 2005–2010	National database	Athletes (competitive or recreational)	15 – 34 years; 0% male	—	—	15 – 24 years: 0.039, 25 – 34 years: 0.047 per 100,000 participants per year	

Study; year	Country; years	Source	Population	Age; sex	Ethnicity	Sport	SrSCD incidence	Pathological findings
Maron et al. [11]; 2013	Minnesota, USA; 1986–2011	US National Registry of Sudden Death in Athletes	Athletes (high school)	12 – 18 years median 16 years); 100% male	100% White	Basketball (n = 5), wrestling (n = 4), cross-country running (n = 3), football (n = 1)	0.68 per 100,000 participants per year (*n* = 13)	SADS (n = 4), CAA (n = 4), HCM (n = 2), ARVC (n = 1), Myocarditis (n = 1), undetermined (n = 1)
Risgaard et al. [12]; 2014	Denmark; 2007–2009	Danish National Patient Registry	Athletes (competitive and non-competitive)	12 – 35 years, 21 ± 8 years; 89% male	—	Running (n = 5), football (n = 2), squash (n = 1), dancing (n = 1)	Non-competitive: 0.43, competitive: 0.47 per 100,000 athletes per year (*n* = 9)	ARVC (n = 3), SUD (n = 3), CAD (n = 1), CHD (n = 1), valve disease (n = 1)
Roberts and Stovitz [13]; 2013	Minnesota, USA; 1993–2012	Minnesota State High School League participation records	Athletes (high school)	12 – 19 years; 100 % male	—	Cross country (n = 2), basketball (n = 1), wrestling (n = 1)	0.24 per 100,000 athletes per year (*n* = 4)	
Suarez-Mier et al. [14]; 2013	Spain; 1995–2010	National Institute of Toxicology and Forensic Sciences of Madrid	Athletes (recreational)	10 – 35 years; —	—	—	(*n* = 80)	CAD (n = 11), ACM (n = 12), HCM (n = 8), ILVH (n = 7), unexplained (n = 18), other (n = 24), 100% underwent autopsy

Study; year	Country; years	Source	Population	Age; sex	Ethnicity	Sport	SrSCD incidence	Pathological findings
Wisten et al. [15]; 2019	Sweden; 2000–2010	Swedish Cause of Death registry, forensic medicine database	General (34% athletes, 66% non-athletes)	10 – 35 years; 90% male	—	Running (n = 11), floor ball (n = 10)	Between 0.1 and 0.6 per 100,000 persons per year (*n* = 62)	SADS (n = 15), HCM (n = 10), ARVC (n = 8), CAD (n = 7), myocarditis (n = 7), other (n = 15), 97% underwent autopsy

Abbreviations: ACM, Arrhythmogenic Cardiomyopathy; ARVC, Arrhythmogenic Right Ventricular Cardiomyopathy; CAA, Coronary Artery Anomalies; CAD, Coronary Artery Disease; CHD, Congenital Heart Disease; CM, Cardiomyopathy; HCM, Hypertrophic Cardiomyopathy; ILVH, Idiopathic Left Ventricular Hypertrophy; SADS, Sudden Arrhythmic Death Syndrome; SUD, Sudden Unexplained Death.

Table S2 Sport-related sudden cardiac arrest incidence for adolescents and young adults (10 – 35 years).

Study; year	Country; years	Source	Population	Age; sex	Ethnicity	Sport	SrSCA incidence	Pathological findings
Bohm et al. [16]; 2021	Germany; 2012–2018	Web-based platform, media monitoring, German resuscitation registry and forensic institutes	Athletes (98% non-elite and recreational)	10–35 years; —	—	—	10–25 years: 0.11, 25–35 years: 0.14 per 100,000 persons per year (*n* = 90, 29 survived)	CAD (n = 13), idiopathic VF (n = 13), myocarditis (n = 11), CM (n = 9), SADS (n = 5) unresolved (n = 34), other (n = 6)

Study; year	Country; years	Source	Population	Age; sex	Ethnicity	Sport	SrSCA incidence	Pathological findings
Bohm et al. [17]; 2023	Germany and Paris area; 2012–2019	Web-based platform, media monitoring, German resuscitation registry and forensic institutes, Paris Sudden Death Expertise Center	Athletes (91.7% recreational)	18–35 years, 28.1 ± 4.8 years; 95.2% male	—	Football (n = 68) and running (n = 35)	0.48 per 100,000 persons per year (*n* = 147, 56 survived)	Of 89 ascertained cases: CAD (n = 23), idiopathic VF (n = 12), myocarditis (n = 10), HCM (n = 7), SADS (n = 6), other (n = 31)
Drezner et al. [18]; 2019	USA; 2014–2016	National Center for Catastrophic Sports Injury Research or UW Medicine Centre for Sports Cardiology, media monitoring	Professional (n = 1), former competitive (within 1 year) (n = 10) and competitive high (n = 78), middle school (n = 28), and college (n = 15) athletes	11–27 years (mean 16 years); 84% male	51% white non-Hispanic/Latino, 30% black/African American, and 11% white Hispanic/Latino, 4% unknown, 3% Asian, 1% Native American	Basketball (30%), American football (25%), track/cross-county (12%) and football (11%)	(*n* = 132, 64 survived)	—
Isern et al [19]; 2023	Norway; 2015–2017	Norwegian Cardiac Arrest Registry, media monitoring	—	12–35 years; —	98% Caucasian, 2% Other	—	0.4 per 100,000 persons per year (*n* = 10)	—

Study; year	Country; years	Source	Population	Age; sex	Ethnicity	Sport	SrSCA incidence	Pathological findings
Sado et al. [20]; 2019	Japan; 2008–2015	National database SPIRITS (Stop and Prevent cardIac aRrest Injury, and Trauma in Schools)	Junior high school, high school, and technical college students	Junior high school: 12–15 years; High school\technical college ≥ 15 years; 85 % male	—	Long-distance running (n = 48), football\futsal (n = 25), basketball (n = 23), baseball (n = 21)	Junior high school: 0.217 (*n* = 61); high school/ technical college: 0.389 (*n* = 108) per 100,000 students per year	—
Visanji et al. [21], 2025	Canada (5 provinces, population of 10.9 million); 2016–2020	Canadian Sudden Cardiac Arrest Network & Canadian Resuscitation Outcomes Consortium	General	18–34 years (26.3 ± 3.9); 91.3 % male (n = 21)	—	—	0.3 per 100,000 persons per year (n = 23), n = 16 had a ROSC and n = 14 survival to discharge (only of 22)	—

Abbreviations: CAD, Coronary Artery Disease; CM, Cardiomyopathy; HCM, Hypertrophic Cardiomyopathy; ROSC, Return of Spontaneous Circulation; SADS, Sudden Arrhythmic Death Syndrome; VF, Ventricular Fibrillation.

Table S3 Sudden cardiac death and arrest incidence for adolescents and young adults (10 – 35 years).

Study; year	Country; years	Source	Population	Age; sex	Ethnicity	Sport	SCD/SCA incidence	Pathological findings
Petek et al. [22]; 2024	USA; 2002–2022	National Centre for Catastrophic Injury Research, Parent Heart Watch, NCAA, and insurance claims	Athletes (NCAA)	Mean 20 years; 83% male	59% White, 37% Black, 3% Hispanic, 1% Asian, 1% Native Hawaiian or Pacific Islander	American football (n = 43), basketball (n = 35), track and field (n = 19), football (n = 12)	SCD: 1.57 per 100,000 athletes per year (n = 143), 50% happened during exertion.	Autopsy-negative SUD (n = 23), ILVH \possible CM (n = 20), HCM (n = 15), CAA (n = 10), CM (n = 9), myocarditis (n = 8), CAD (n = 7), other (n = 26), 118 cases had enough information for adjudication
Peterson et al. [23]; 2021	USA; 2014–2018	National Centre for Catastrophic Injury Research, National sports organisations	Athletes (Middle (15.4 %) or high school (61.6 %), college and semi\professional (16.6 %))	11 – 29 years (mean 16.7 years); 83.7% male	49.5% Caucasian, 32.9% African-American, 8.5% Latino/Hispanic, 5.7% Unknown, 2.7% Asian, 0.6% Native American	Basketball (n = 95, 43 survived), American football (n = 84, 34 survived), football (n = 39, 22 survived), track\cross country (n = 31, 17 survived)	SCA: High school: 1.52, NCAA: 1.97 per 100,000 athletes per year (n = 331, 158 survived, 78% occurred during or within one h of exercise cessation)	HCM (n = 43), ILVH (n = 28), CAA (n =25), AN-SUD (n = 20), CM (n = 13), myocarditis (n = 9), other (n = 71); (n = 209 had an aetiology confirmed).

Abbreviations: AN-SUD, Autopsy-Negative Sudden Unexplained Death; CAA, Coronary Artery Anomalies; CAD, Coronary Artery Disease; CM, Cardiomyopathy; HCM, Hypertrophic Cardiomyopathy; ILVH, Idiopathic Left Ventricular Hypertrophy; NCAA, National Collegiate Athletic Association; SUD, Sudden Unexplained Death

Table S4 Explainable artificial intelligence addressing life-threatening arrhythmias.

Study; year	Data type	Data source	Objective	AI model	AI performance	xAI method
Abousaber, El-Ghaish, and Abdallah [24]; 2025	48, 30 min-long, ECGs from lead II	Publicly available dataset: MIT-BIH arrhythmia database	Multiple heartbeat classification	VAE & CNN	Accuracy: 0.996, F1-score: 0.954	SHAP & Grad-CAM
Ahn et al. [25]; 2024	1,889 cardiac arrest ECGs from lead II (12.2 % VF, 1.7 % pVT)	Private dataset	Predict shockable rhythms from ECGs with compression artefacts	CNN	Accuracy: 0.749, AUPRC: 0.870, AUROC: 0.867, NPV: 0.893, PPV: 0.664, sensitivity: 0.914, specificity: 0.610	Grad-CAM (heatmap)
Ali et al.[26]; 2025	48, 30 min-long, ECGs from lead II	Publicly available dataset: MIT-BIH arrhythmia database	Multiple heartbeat classification	CNN	Accuracy: 0.990, AUROC: 1.000, F1-score: 0.910, NPV: 0.893, precision: 0.900, sensitivity: 0.930, specificity: 0.990	Sig LIME
Alqudah and Alfraihat [27]; 2025	48, 30 min-long, ECGs from lead II and 448 ECGs from 12-leads	Publicly available dataset: MIT-BIH arrhythmia database and PTB diagnostic ECG	Multiple heartbeat classification	CNN & LSTM	Accuracy: 0.932, F1-score: 0.929, sensitivity: 0.928, specificity: 0.930	SHAP & Grad-CAM

Study; year	Data type	Data source	Objective	AI model	AI performance	xAI method
Ayano et al. [28]; 2024	—	Publicly available dataset: PTB-XL, CODE-15%, and the reduced seven and merged four classes of the Chapman Arrhythmia dataset	Multiple arrhythmia classification	CNN & LSTM	PTB-XL: Accuracy: 0.899, F1- score: 0.761, specificity: 0.935; CODE-15%: Accuracy: 0.978; Reduced Chapman Accuracy: 0.986; Merged Chapman: Accuracy:0 .988	SHAP & Grad-CAM++
Cao et al. [29]; 2022	448 ECGs from 12-leads	Publicly available dataset: PTB diagnostic	MI detection and localisation	RNN	MI detection: Accuracy: 0.998, sensitivity: 0.994, specificity: 0.998; MI localisation: Accuracy: 0.998, sensitivity: 0.988, specificity: 0.998.	Grad-CAM (Polar plots of lead importance)
Chu et al. [30]; 2025	48, 30 min-long, ECGs from lead II	Publicly available dataset: MIT-BIH arrhythmia database	Multiple heartbeat classification	GCN	Accuracy: 0.986	Graph visualisation
Fiorina et al. [31]; 2025	247,254 14-day ambulatory single-lead ECG recordings. 1,104 had sustained VT or VF	Private dataset from 6 countries (USA, UK, France, Czech Republic, South Africa, and India).	VT & VF low or high-risk prediction (in the following 13 days, based on the likelihood of the first 24h)	CNN	AUROC: 0.948, sensitivity: 0.661, specificity: 0.970;	Saliency maps
Ganeshkumar et al. [32]; 2023	6,877, 6-s to 60-s-long ECGs from leads aVF, V5, and V6	Publicly available dataset: CPSC 2018	Multiple arrhythmia classification	CNN	Accuracy: 0.962, F1-score: 0.967, Hamming loss: 0.037, precision: 0.986, sensitivity: 0.949	Grad-CAM (heatmap)

Study; year	Data type	Data source	Objective	AI model	AI performance	xAI method
Habib et al. [33]; 2023	48, 30 min-long, ECGs from lead II	Publicly available dataset: MIT-BIH arrhythmia database	Multiple heartbeat classification	CNN with sinc-convolution layer	Accuracy: 0.918	Vector-based joint time-frequency representation
Hammachi et al. [34]; 2022	48, 30 min-long, ECGs from lead II	Publicly available dataset: MIT-BIH arrhythmia database	Multiple heartbeat classification	CNN + LSTM	CNN**:** Accuracy: 0.971, F1-score: 0.936, precision: 0.900, sensitivity: 0.988; LSTM: Accuracy: 0.985, F1-score: 0.961, precision: 0.940, sensitivity: 0.988	Permutation feature importance and Grad-CAM
Huang et al. [35]; 2024	—	Publicly available dataset: PTB-XL, Georgia, Chapman & CPSC 2018, and CinC 2017	Multiple arrhythmia classification (STC, PVC, WPW, and AF)	CNN	F1- score: 0.776 (STC); 0.885 (PVC); 0.825 (WPW); 0.854 (AF)	SHAP
Jin et al. [36]; 2022	48, 30 min-long, ECGs from lead II, and 6,877, 6 s to 60 s-long, ECGs from 12 leads	Publicly available dataset: MIT-BIH arrhythmia database & CPSC 2018	Multiple arrhythmia classification	CNN	MIT: Accuracy: 0.888, F1-macro: 0.810; CPSC: Accuracy: 0.601, F1-macro: 0.653	Attention mechanism weights

Study; year	Data type	Data source	Objective	AI model	AI performance	xAI method
Jo et al. [37]; 2021	Training: 86,802, 10-s-long ECGs from 12 leads; testing: 36,961 10-s-long ECGs from 12 leads	Publicly available dataset: PTB-XL, Georgia, Chapman & CPSC 2018 (testing); privately available database: Sejong dataset (training)	Multiple arrhythmia classification	NN-backed ensemble tree	AUROC: 0.966, F1-score: 0.844, precision: 0.914, sensitivity: 0.795	Saliency\sensitivity map
Kolk et al. [38]; 2024	32,129 ICD ECGs from 2,942 patients (61.7 ± 13.9 years), with a mean follow-up of 43.9 ± 35.9 years	Private dataset: Multicentre study from two academic hospitals between 2007– 2021	VT & VF classification	VAE & RF	AUROC: 0.738	Saliency map
Kovalchuk et al. [39]; 2025	—	Publicly available dataset: MIT-BIH arrhythmia database, QT database, CPSC 2020 database, and University of Glasgow database.	Multiple arrhythmia classification	CNN	MIT: Accuracy: 0.999, F1-score: 0.989, precision: 0.992, sensitivity: 0.987; QT: Accuracy: 0.999, F1-score: 0.982, precision: 0.980, sensitivity: 0.982; CPSC: Accuracy: 0.999, F1-score: 0.990, precision: 0.986, sensitivity: 0.994; Glasgow: Accuracy: 0.999, F1-score: 0.924, precision: 0.983, sensitivity: 0.901	Saliency maps

Study; year	Data type	Data source	Objective	AI model	AI performance	xAI method
Le et al. [40]; 2023	Training: CPSC 2018: 6,877 6-s to 60-s-long ECGs, Chapman: 10,646 10-s-long ECGs. For both datasets, only leads I, II, and V1 are used	Publicly available dataset: Chapman & CPSC 2018	Multiple arrhythmia classification	CNN	Chapman: Accuracy: 0.987, F1-score: 0.972, precision: 0.974, sensitivity: 0.970; CPSC-2018: Accuracy: 0.963, F1-score: 0.800, precision: 0.821, sensitivity: 0.786	Grad-CAM (heatmap)
Li et al. [41]; 2025	—	Publicly available dataset: MIT-BIH arrhythmia database, MIT-BIH supraventricular database, INCART database, and SCD-Holter database	Multiple arrhythmia classification	CNN	MIT-Sup: Accuracy: 0.511, AUROC: 0.628, F1-score: 0.565, precision: 0.570, sensitivity: 0.560; MIT-Arr: Accuracy: 0.768, AUROC: 0.819, F1-score: 0.795, precision: 0.832, sensitivity: 0.762; INCART: Accuracy: 0.894, AUROC: 0.928, F1-score: 0.898, precision: 0.919, sensitivity: 0.877; SCD: Accuracy: 0.614, AUROC: 0.647, F1-score: 0.651, precision: 0.694, sensitivity: 0.611	Interpretability-driven attention constraint mechanism.

Study; year	Data type	Data source	Objective	AI model	AI performance	xAI method
Liu et al. [42]; 2025	19,759 8-lead ECG records with the 20 most common ECG categories, sampled at 500 Hz.	Publicly available dataset: Tianchi ECG dataset	Multiple arrhythmia classification	Transformer	Micro F1- score: 0.916, precision: 0.943, sensitivity: 0.890	Attention-based heatmap
Lu et al. [43]; 2024	2,315,782 7-s to 10-s-long ECGs from 12 leads	Private dataset: Telehealth centre in Minas Gerais	Multiple arrhythmia classification	DNN	AUROC: 0.998, F1-score: 0.948	Feature importance through isolation strategy (heatmaps)
Mamun et al. [44]; 2025	29 paediatric and 10 adult patients with congenital heart disease, totalling 113,924 labelled beats across 20 arrhythmia categories	Private dataset: Leipzig dataset	Multiple arrhythmia classification (AVNRT, AVRT, AFL)	CNN with a transformer block	F1- score: 0.912, precision: 0.934,	Attention heatmap and SHAP

Study; year	Data type	Data source	Objective	AI model	AI performance	xAI method
Nankani et al. [45]; 2021	—	Publicly available dataset: MIT-BIH arrhythmia database, Malignant ventricular arrhythmia database, Creighton University Ventricular Tachyarrhythmia database, Ideo ventricular arrhythmia database; private database: AHA	VT & VF classification	RNN	Accuracy: 0.981, sensitivity: 0.962, specificity: 0.986,	Gradient-based guided backpropagation (saliency maps)
Neves et al. [46]; 2021	48, 30 min-long, ECGs from lead II	Publicly available dataset: MIT-BIH arrhythmia database	Multiple heartbeat classification	CNN + kNN	CNN: AUROC: 0.886, F1-score: 0.861, precision: 0.926, sensitivity: 0.818; kNN: AUROC: 0.707, F1-score: 0.705, precision: 0.865, sensitivity: 0.611	Permutation sample importance, SHAP, and LIME
Pasero, Casella, and Randazzo [47]; 2025	1,501, 6-s to 60-s-long ECGs, 12-leads	Publicly available dataset: CPSC 2018	Brugada syndrome classification	CNN & LSTM	F1-score: 0.950 (Brugada-like), 0.920 (Non-Brugada), 0.890 (Ambiguous class)<br>Precision: 0.940 (Brugada-like), 0.910 (Non-Brugada), 0.880 (Ambiguous class);<br>Sensitivity: 0.960 (Brugada-like), 0.930 (Non-Brugada), 0.900 (Ambiguous class);	Attention heatmaps

Study; year	Data type	Data source	Objective	AI model	AI performance	xAI method
Potharaju et al. [48]; 2025	48, 30 min-long, ECGs from lead II	Publicly available dataset: MIT-BIH arrhythmia database	Multiple heartbeat classification	CNN + LSTM	F1-score: 0.992 (CNN), 0.970 (LSTM)	Grad-CAM
Shylaja and Kumari [49]; 2025	48, 30 min-long, ECGs from lead II	Publicly available dataset: MIT-BIH arrhythmia database	Multiple heartbeat classification	CNN	Accuracy: 0.993, F1- score: 0.983, precision: 0.984, sensitivity: 0.982	Attention heatmaps
Tabatabaei et al. [50]; 2024	MIT: 48, 30 min-long, ECGs from lead II AF: 7,130 PVC QRS complexes, and 75,048 non-PVC QRS complexes, subjects aged 23 and 24 h ECG signals obtained from 84 subjects	Publicly available dataset: MIT-BIH arrhythmia database and long-term AF database	Multiple heartbeat classification	CNN & LSTM	Accuracy: 0.982, sensitivity: 0.861, specificity: 0.975	SHAP
Tao et al. [51]; 2024	PTB-XL: 21,837, 10 s-long, ECGs from 12 leads; Hefei: 20,036, 10 s-long, ECGs from leads I, II, and V1–V6; Lobachevsky: 200, 10 s-long, ECGs from 12-leads	Publicly available dataset: PTB-XL, Hefei Hi-tech cup ECG intelligent competition, and Lobachevsky University Electrocardiography database	Multiple heartbeat classification	DNN	PTB-XL: Accuracy: 0.892, AUROC: 0.929, F1-score: 0.770, precision: 0.752, sensitivity: 0.790; Hefei: AUROC: 0.981, F1-score: 0.809, precision: 0.880, sensitivity: 0.768; Lobachevsky: AUROC: 0.998, F1-score: 0.989, precision: 0.992, sensitivity: 0.986	GradCAM (heatmap)

Study; year	Data type	Data source	Objective	AI model	AI performance	xAI method
Xu et al. [52]; 2022	6,877, 6-s to 60-s-long ECGs from 12 leads	Publicly available dataset: CPSC 2018	Multiple arrhythmia classification	RNN	F1-score: 0.831	SHAP (lead-importance)
Zhang et al. [53]; 2021	6,877, 6-s to 60-s-long ECGs from 12 leads	Publicly available dataset: CPSC 2018	Multiple arrhythmia classification	CNN	Accuracy: 0.966, AUROC: 0.970, F1-score: 0.813, precision: 0.821, sensitivity: 0.812	SHAP (lead-importance)

Abbreviations: AF, Atrial Fibrillation; AFL Atrial Flutter; AUROC, Area Under the Receiver Operating Characteristic Curve; AUPRC, Area Under the Precision-Recall Curve; AVNRT, Atrioventricular Nodal Reentrant Tachycardia; AVRT, Atrioventricular Reentrant Tachycardia; CNN, Convolutional Neural Network; DNN, Deep Neural Network; ECG, Electrocardiogram; Grad-CAM, Gradient-weighted Class Activation Mapping; GCN, Graph Convolutional Network; ICD, Implantable Cardiac Defibrillator; kNN, k-Nearest Neighbours; LIME, Local Interpretable Model-agnostic Explanations; LSTM, Long Short-Term Memory; MI, Myocardial Infarction; NN, Neural Network; NPV, Negative Predictive Value; PPV, Positive Predictive Value; PVC, Premature Ventricular Contraction; pVT, Polymorphic Ventricular Tachycardia; RF, Random Forest; RNN, Recurrent Neural Network; SHAP, Shapley Additive Explanations; STC, ST-changes; VAE, Variational Autoencoder; VF, Ventricular Fibrillation; VT, Ventricular Tachycardia; WPW, Wolff-Parkinson-White Syndrome.

Table S5 Cardiac electrophysiological models.

Study; year	Model focus	Model objective	Model derivation	Model novelty	Model disadvantage
Ai et al. [54]; 2020	Whole-heart electrophysiology	Time-critical and dynamic physiological-based computational heart model	—	The heart model is constructed from discrete hybrid automaton models	Simplistic hybrid automaton model that is amenable to formal analysis and real-time emulation
Alaa et al. [55]; 2014	Cardiac electrophysiology	Analysis of the propagation of ionic concentrations and electrical potentials in space and time	Nernst-Planck flux equation	New cable equation by assuming the membrane acts as an electrical circuit in which resistance and capacitance are arranged in a parallel circuit	Simplified model
Biasi et al. [56]; 2021	Ventricular electrophysiology	Human ventricular epicardial cells and their reentry dynamics. The total ionic current is divided into excitatory, recovery, and transient outward currents	Rogers-McCulloch formulation of the FitzHugh-Nagumo equations	First phenomenological model to resemble human experimental data with three state variables and 17 parameters	The model simulations are restricted to epicardial cells
Bucelli et al. [57]; 2022	Fully-coupled heart	3D description of cardiac electrophysiology, active and passive mechanics, and haemodynamics, together with a closed-loop lumped-parameter model to simulate systemic and pulmonary circulation	—	Accounts for the major feedback effects, including electro-mechanical and mechano-electrical feedback as well as force-strain and force-velocity relationships	The left atrium is modelled as passive from both the electrical and mechanical viewpoints
Chen et al. [58]; 2024	Fully-coupled heart	Boost the real-time efficiency of the cardiac electromechanical simulation	—	Separate the process into cell-level electrical stimulation and organ-level electrical stress diffusion/conduction	Simplified model by using mechanical isotropic fibres for the myocardial electromechanical coupling

Study; year	Model focus	Model objective	Model derivation	Model novelty	Model disadvantage
Collin et al. [59]; 2015	State estimation	Joint state and parameter observer, where a Luenberger observer corrects the state, and a reduced-order optimal Kalman-based filter is restricted to the conductivity parametric space	1D monodomain and surface-based bidomain model	Using a Luenberger observer for reaction-diffusion models with propagating front features	Only an approximation of the real state
Cusimano and Gerardo-Giorda [60]; 2018	Cardiac electrophysiology	Account for the effects of high structural heterogeneity in the spatio-temporal description of excitation waves propagation	Monodomain equation	Replace the diffusive term with a fractional power of the monodomain equation	Computational cost due to the very fine spatial grids needed for the space-fractional operator
Del Corso et al. [61]; 2022	Whole-heart electrophysiology	Reproduce the cardiac electrophysiology of the whole heart in healthy and bundle branch block conditions	Bidomain model together with the Courtemanche (atrial myocytes), Stewart (Purkinje network), and ten Tusscher-Panfilov model (ventricular myocytes)	Decomposition of whole cardiac geometry into a set of coupled conductive media of different topology: atrial myocytes, Purkinje network, and ventricular myocytes	Computationally expensive
Delestri et al. [62]; 2025	Ventricular electromechanics	Investigate the significance of cardiac induced-coronary arterial motion on coronary haemodynamic	—	Coupling a 3D biventricular electromechanics model with a 3D coronary artery model	The model excludes the physical representation of the atrial chambers

Study; year	Model focus	Model objective	Model derivation	Model novelty	Model disadvantage
Elkaranshawy et al. [63]; 2021	Whole-heart electrophysiology	Developing a mathematical model that represents the interactions between different pacemakers and allows analysis of cardiac rhythms	Van der Pol and FitzHugh-Nagumo equations	Incorporation of heart rate variability phenomenon, respiratory sinus arrhythmia and Mayer waves	The parameters calculation is not accurate enough to represent the real phenomena
Fedele et al. [64]; 2023	Fully-coupled heart	A biophysically detailed electromechanical model of the whole human heart that considers both atrial and ventricular contraction	—	Inclusion of atrial contraction	Transmural heterogeneity of ionic models is not considered. Furthermore, the mesh size is not fine enough to model pathological scenarios
Georgieva-Tsaneva [65]; 2025	Cardiac electrophysiology	Simulation of ECG signals	Gaussian mesa function (morphology) and Rössler attractor (RR-interval variability)	Integrate accurately model waveform morphology and heart rate variability	A deterministic model is not capable to emulate stochasticity observed in real heart rate variability dynamics
Gilette et al. [66]; 2021	Ventricular electrophysiology	His-Purkinje modelling for cardiac digital twinning pipelines	—	Two-stage approach for His-Purkinje personalisation	Computational costs of the His-Purkinje system construction and simulation

Study; year	Model focus	Model objective	Model derivation	Model novelty	Model disadvantage
Hazim et al. [67]; 2019	Electromechanical model of cardiac tissue	Model effect of mechano-electrical feedback on the action potential duration for a range of basic cycle lengths to illustrate the effects on the onset of cardiac alternans	Monodomain equation with Luo-Rudy I membrane model	Inclusion of stretch-activated currents induced by stretch-activated ion channels	The assumption that voltage instability causes cardiac alternans (as opposed to calcium-driven instability, for example)
Hurtado and Kuhl [68]; 2014	Ventricular electrophysiology	Study regional and temporal variations in both ventricular depolarisation and repolarisation by using the concept of non-uniform refractoriness	FitzHugh-Nagumo (electrical response) and Aliev-Panfilov (biochemical response) model	Reinterpret a phenomenological scaling parameter as the refractoriness, and establish a functional relation between the local action potential and this material parameter	The model is solely validated for the left ventricle with ten averaged clinically measured ECGs
Jackowska-Zduniak [69]; 2022	AVNRT	Recreate pathological AVNRT behaviour through a system of stochastic differential equations	Van der Pol equation	Introduction of a stochastic term allows modelling a pre-excitation perturbation, PAC or PVC	Stochastic perturbation over-models the AVNRT arrhythmia phenomenon in particular cases
Jacquemet [70]; 2016	Cardiac electrophysiology	Simple model of exponential-shaped repolarisation in an electrotonically-coupled cell network with different intrinsic action potential duration	—	The model is amenable to analytical calculations for both the forward and inverse problems, besides enabling the study of the existence and uniqueness of the solution to the inverse problem	Formulation of the simplified cell model and the particular choice of action potential duration

Study; year	Model focus	Model objective	Model derivation	Model novelty	Model disadvantage
Jæger et al. [71]; 2021	Whole-heart electrophysiology	The extracellular, membrane, and intracellular spaces are represented as individual cells in the model (called the EMI model), instead of being averaged as in the bidomain model	Bidomain model	The approach allows for detailed analysis of the biophysical processes going on in functionally important spaces very close to individual myocytes	Significant computational increase
Jæger and Tveito [72]; 2023	Whole-heart electrophysiology	Kirchhoff network model, a model which represents each myocyte and allows manipulation at the cellular level, and between cells, but not within individual cells	EMI model from [71]	Applying Kirchhoff's current law to reduce computational costs	Less accuracy compared to the EMI model
Johnston and Johnston [73]; 2013	Whole-heart electrophysiology	A mathematical model to retrieve all six cardiac bidomain conductivity parameters in a block of cardiac tissue through a multi-electrode array	Bidomain equation	Retrieving six rather than previously four conductivity parameters	Theoretical framework based on simulations
Labarthe et al. [74]; 2014	Atrial electrophysiology	Bilayer model of the human atria capable of recreating recorded activation patterns	Monodomain equation	Fully capturing propagation patterns seen in vivo, which is not possible in tissue or surface models	The model is suited to examine global behaviour over long periods and would probably fail to capture fine features of local propagation
Lee et al. [75]; 2023	Myocardial growth	A three-dimensional constitutive model that describes an electro-visco-elastic growth response on the myocardium	—	The growth direction for each element is selected at each time step based on the mechanical conditions	No consideration of the mechano-electric feedback

Study; year	Model focus	Model objective	Model derivation	Model novelty	Model disadvantage
Meisenzahl et al. [76]; 2024	Cardiac electrophysiology	Locate the origin of the QRS complex from 12-lead ECGs in the presence of a scar issue	BOATMAP (Bayesian Optimisation Active Targeting for Monomorphic Arrhythmia Pace mapping)	Real-time interpretable guidance for accurate localisation of ventricular activation	Tested on limited heart geometries and no exploration of variations in the His-Purkinje system.
Moss et al. [77]; 2021	Fully-coupled heart	Human 4-chamber electro-mechanically coupled whole heart in silico model embedded within a torso model	—	Analysis of the heart's contraction and its influences on the ECG	Difficulty in generalising results since the scope of the work depends on the used geometry
Pagani et al. [78]; 2021	AF	Numerically predict paroxysmal and persistent forms of AF by showing how substrate characteristics play a key role in inducing and sustaining these arrhythmias	Monodomain and Courtemanche-Ramirez-Nattel model	New way of setting a parametrisation of Electrophysiological mathematical models based on conduction velocities estimated from electro-anatomical maps during NSR of two patients	Approach of using structural causes limits the model in reproducing functional causes of AF, such as the cellular remodelling in human hearts affected by AF
Passow and Tenderich [79]; 2025	Cardiac electrophysiology	Mathematically demonstrate R to T-wave concordance of the human ECG	—	3-dimensional, time-dependent equation simulating the transmural gradient	Simplified model without atrial activity

Study; year	Model focus	Model objective	Model derivation	Model novelty	Model disadvantage
Podziemski and Zebrowski [80]; 2013	Atrial electrophysiology	Simplification of the right atrium to a two-dimensional plane and a cylindrical surface	Van der Pol and FitzHugh-Nagumo \ Fenton-Karma equation	Inclusion of the nodal tissue into the same model, together with the atrial muscle tissue	Simplified model
Quiroz-Juárez et al. [81]; 2018	Cardiac electrophysiology	12-lead-ECG signal generation with realistic heart rate dynamics	Van der Pol equation and the FitzHugh-Nagumo equation	Inclusion of heart rate variability	The assumption that depolarisation and repolarisation waves are described as separate processes
Rabinovitch [82]; 2021	Heart fibrosis electrophysiology	A cellular automaton model to analyse percolation and transport of a distributed-fibrosis-inflicted heart-like tissue	Cellular automata	Analysis of transport, tortuosity and percolation on action potential in heart fibrosis	Simplicity of the model
Romitti et al. [83]; 2025	Atrial electrophysiology	Replicate atrial electrophysiology at different AF stages, including persistent AF, with a phenomenological model instead of biophysical models	Cellular automata	Extend the ventricular cellular automaton to atrial electrophysiology	Moderate sensitivity compared to biophysical models
Sabzpoushan and Ghajarjazy [84]; 2020	Ventricular electrophysiology	A three-state variable model to research the impact of notch-dome dynamics on the heart function	—	Simulation of notch-dome dynamics and morphology through a novel three-state variable model	Disregard of action potential dynamics like action potential propagation and restitution

Study; year	Model focus	Model objective	Model derivation	Model novelty	Model disadvantage
Salvador et al. [85]; 2021	Ventricular electromechanics	Patient-specific computational model for the numerical simulation of ventricular electromechanics in patients with ischemic cardiomyopathy for NSR and VT	Tuscher-Panfilov ionic model	Synthesise both electrical and mechanical activities into a cohesive framework	Solely considered the left ventricle for simulations
Tenderini et al. [86]; 2022	Cardiac electrophysiology	Estimate the epicardial potential from measurements of the electrical potential at a discrete set of points on the body surface	—	PDE-aware deep learning model for the numerical solution to the inverse problem of electrocardiography	Simplified dataset (in silico data) while using the same reference geometry, i.e., ignoring geometrical variability
Trovato et al. [87]; 2020	AF	Study the role of the myocardium with fractal characteristics, on atrial fibrillatory dynamics	—	Action potential propagation is based on complex fractal order derivatives	Simulations are performed in 2D domains
Ugarte et al. [88]; 2018	AF	Study the role of the effect of structurally heterogeneous tissue on atrial fibrillatory dynamics	Courtemanche atrial membrane formalism	Mathematical model based on complex fractional order derivatives	Simulations are performed in 2D domains, although the atrial tissue is 3D and atrial heterogeneities impact rotor dynamics.
Wallman and Sandberg [89]; 2018	AV-node electrophysiology	Model of the human AV-node, consisting of a series of interacting nodes, each with separate dynamics in refractory time and conduction delay. Simulated on NSR and AF	Network model	First model to capture both spatial and temporal dynamics while being efficient enough to allow interactive use on clinical timescales, as well as parameter estimation and uncertainty quantification	Especially during AF simulation, the assumption is that both slow and fast pathways are simultaneously activated by atrial impulses.

Study; year	Model focus	Model objective	Model derivation	Model novelty	Model disadvantage
Yang et al. [90]; 2025	Whole-heart electrophysiology	Compute an ECG and body surface potential maps	Biodomain model with Fitzhugh-Nagumo model	Using a realistic heart-torso to include the effects of the heart position relative to the torso on the 12-lead ECG	T-wave heterogeneity, since both ventricular depolarisation and repolarisation extended from the endocardium to the myocardium (the T-wave normally starts from the epicardium to the endocardium).
Zappon et al. [91]; 2024	Fully-coupled heart	Simulation of ECGs and body surface potential maps by coupling a cardiac electromechanical model during NSR and VT	Monodomain model coupled with the ten Tusscher-Panfilov ionic model	Inclusion of the torso domain deformation induced by the myocardial displacement	Electromechanical coupling lacks the representation of the stretch-activated channels

Abbreviations: AF, Atrial Fibrillation; AVNRT, Atrioventricular Node Reentrant Tachycardia; ECG, Electrocardiogram; EMI, Extracellular-Membrane-Intracellular; NSR, Normal Sinus Rhythm; PAC, Premature Atrial Contractions; PDE, Partial Differential Equation; PVC, Premature Ventricular Contractions; VT, Ventricular Tachycardia.

Table S6 Prediction model Risk Of Bias ASsessment Tool (PROBAST) for the 89 papers meeting the inclusion criteria.

**Author; year**	**Risk of Bias**				**Applicability**			**Overall**	
	Participant	Predictors	Outcome	Analysis	Participant	Predictors	Outcome	**Risk of Bias**	**Applicability**
Abousaber, El-Ghaish, and Abdallah [24]; 2025	+	+	+	+	+	+	+	+	+
Ahn et al. [25]; 2024	+	+	+	+	+	+	+	+	+
Ai et al. [54]; 2020	?	+	+	+	?	+	+	+	+
Alaa et al. [55]; 2014	?	+	+	+	?	+	+	+	+
Ali et al.[26]; 2025	+	+	+	+	+	+	+	+	+
Alqudah and Alfraihat [27]; 2025	+	+	+	+	+	+	+	+	+
Ayano et al. [28]; 2024	+	+	+	+	+	+	+	+	+

Author; year	Risk of Bias				Applicability			Overall	
	Participant	Predictors	Outcome	Analysis	Participant	Predictors	Outcome	**Risk of Bias**	**Applicability**
Biasi et al. [56]; 2021	?	+	+	?	+	+	+	?	?
Boden et al. [8]; 2013	+	+	-	+	+	+	?	+	+
Bohm et al. [16]; 2021	+	?	+	?	+	?	?	?	?
Bohm et al. [17]; 2023	+	?	+	+	+	?	+	+	+
Bucelli et al. [57]; 2022	?	+	+	+	?	+	+	+	+
Cao et al. [29]; 2022	+	+	+	+	+	+	+	+	+
Chen et al. [58]; 2024	+	+	+	+	+	+	+	+	+
Chu et al. [30]; 2025	+	+	+	+	+	+	+	+	+
Collin et al. [59]; 2015	?	+	+	+	?	+	+	+	+

Author; year	Risk of Bias				Applicability			Overall	
	Participant	Predictors	Outcome	Analysis	Participant	Predictors	Outcome	**Risk of Bias**	**Applicability**
Cusimano and Gerardo-Giorda [60]; 2018	?	+	+	+	?	+	+	+	+
Del Corso et al. [61]; 2022	?	+	+	?	?	+	+	+	+
Delestri et al. [62]; 2025	+	+	+	+	+	+	+	+	+
Drezner et al. [18]; 2019	+	+	+	+	+	+	+	+	+
Elkaranshawy et al. [63]; 2021	?	+	+	+	?	+	+	+	+
Fedele et al. [64]; 2023	?	+	+	?	?	+	+	+	+
Fiorina et al. [31]; 2025	+	+	+	+	+	+	+	+	+
Ganeshkumar et al. [32]; 2023	+	+	+	+	+	+	+	+	+

Author; year	Risk of Bias				Applicability			Overall	
	Participant	Predictors	Outcome	Analysis	Participant	Predictors	Outcome	**Risk of Bias**	**Applicability**
Georgieva-Tsaneva [65]; 2025	?	+	+	+	+	+	+	+	+
Gilette et al. [66]; 2021	?	+	+	-	?	+	+	-	-
Ha et al. [9]; 2022	+	+	+	+	+	+	+	+	+
Habib et al. [33]; 2023	+	+	+	+	+	+	+	+	+
Hammachi et al. [34]; 2022	+	+	+	+	+	+	+	+	+
Hazim et al. [67]; 2019	?	+	+	+	?	+	+	+	+
Huang et al. [35]; 2024	+	+	+	-	+	+	+	?	+
Hurtado and Kuhl [68]; 2014	?	+	+	+	?	+	+	+	+

Author; year	Risk of Bias				Applicability			Overall	
	Participant	Predictors	Outcome	Analysis	Participant	Predictors	Outcome	**Risk of Bias**	**Applicability**
Isern et al [19]; 2023	+	+	?	?	+	+	?	?	?
Jackowska-Zduniak [69]; 2022	-	+	+	-	-	+	+	-	-
Jacquemet [70]; 2016	?	?	+	?	?	+	+	?	?
Jæger et al. [71]; 2021	-	+	+	-	-	+	+	-	-
Jæger and Tveito [72]; 2023	?	+	+	+	?	+	+	+	+
Jin et al. [36]; 2022	+	+	+	+	+	+	+	+	+
Jo et al. [37]; 2021	+	+	+	+	+	+	+	+	+

Author; year	Risk of Bias				Applicability			Overall	
	Participant	Predictors	Outcome	Analysis	Participant	Predictors	Outcome	**Risk of Bias**	**Applicability**
Johnston and Johnston [73]; 2013	?	+	+	+	?	+	+	+	+
Kolk et al. [38]; 2024	+	+	+	-	+	+	+	?	+
Kovalchuk et al. [39]; 2025	+	+	+	+	+	+	+	+	+
Labarthe et al. [74]; 2014	?	+	+	+	?	+	+	+	+
Lee et al. [75]; 2023	+	+	+	+	+	+	+	+	+
Le et al. [40]; 2023	?	+	+	+	?	+	+	+	+
Li et al. [41]; 2025	+	+	+	+	+	+	+	+	+
Liu et al. [42]; 2025	+	+	+	+	+	+	+	+	+

Author; year	Risk of Bias				Applicability			Overall	
	Participant	Predictors	Outcome	Analysis	Participant	Predictors	Outcome	**Risk of Bias**	**Applicability**
Lu et al. [43]; 2024	+	+	+	+	+	+	+	+	+
Mamun et al. [44]; 2025	+	+	+	+	+	+	+	+	+
Marijon et al. [10]; 2013	+	+	+	+	+	+	+	+	+
Maron et al. [11]; 2013	+	+	+	+	+	+	+	+	+
Meisenzahl et al. [76]; 2024	+	+	+	+	+	+	+	+	+
Moss et al. [77]; 2021	?	+	+	-	?	+	+	-	-
Nankani et al. [45]; 2021	+	+	+	+	+	+	+	+	+
Neves et al. [46]; 2021	+	+	+	+	+	+	+	+	+
Pagani et al. [78]; 2021	+	+	+	-	?	+	+	-	-

Author; year	Risk of Bias				Applicability			Overall	
	Participant	Predictors	Outcome	Analysis	Participant	Predictors	Outcome	**Risk of Bias**	**Applicability**
Pasero, Casella, and Randazzo [47]; 2025	+	+	+	+	+	+	+	+	+
Passow and Tenderich [79]; 2025	+	+	+	+	+	+	+	+	+
Petek et al. [22]; 2024	+	+	+	+	+	+	+	+	+
Peterson et al. [23]; 2021	+	+	+	+	+	+	+	+	+
Podziemski and Zebrowski [80]; 2013	-	+	+	-	-	+	+	-	-
Potharaju et al. [48]; 2025	+	+	+	+	+	+	+	+	+
Quiroz-Juárez et al. [81]; 2018	-	+	+	+	+	+	+	+	+

Author; year	Risk of Bias				Applicability			Overall	
	Participant	Predictors	Outcome	Analysis	Participant	Predictors	Outcome	**Risk of Bias**	**Applicability**
Rabinovitch [82]; 2021	-	+	+	-	-	+	+	-	-
Risgaard et al. [12]; 2014	+	+	+	+	+	+	+	+	+
Roberts and Stovitz [13]; 2013	+	+	+	+	+	+	+	+	+
Romitti et al. [83]; 2025	?	+	+	+	+	+	+	+	+
Sabzpoushan and Ghajarjazy [84]; 2020	-	+	+	-	-	+	+	-	-
Sado et al. [20]; 2019	+	+	+	+	+	+	?	+	+
Salvador et al. [85]; 2021	-	+	+	-	-	+	+	-	-
Shylaja and Kumari [49]; 2025	+	+	+	+	+	+	+	+	+

Author; year	Risk of Bias				Applicability			Overall	
	Participant	Predictors	Outcome	Analysis	Participant	Predictors	Outcome	**Risk of Bias**	**Applicability**
Suarez-Mier et al. [14]; 2013	+	+	-	-	+	+	?	-	-
Tabatabaei et al. [50]; 2024	+	+	+	+	+	+	+	+	+
Tao et al. [51]; 2024	+	+	+	+	+	+	+	+	+
Tenderini et al. [86]; 2022	?	+	+	+	?	+	+	+	+
Trovato et al. [87]; 2020	?	+	+	-	?	+	+	-	-
Ugarte et al. [88]; 2018	-	+	+	-	-	+	+	-	-
Visanji et al. [21], 2025	+	+	+	+	+	+	+	+	+
Wallman and Sandberg [89]; 2018	?	+	+	-	?	+	+	-	-

Author; year	Risk of Bias				Applicability			Overall	
	Participant	Predictors	Outcome	Analysis	Participant	Predictors	Outcome	**Risk of Bias**	**Applicability**
Wisten et al. [15]; 2019	+	+	+	-	+	+	+	-	?
Xu et al. [52]; 2022	+	+	+	+	+	+	+	+	+
Yang et al. [90]; 2025	+	+	+	+	+	+	+	+	+
Zappon et al. [91] 2024	-	+	+	-	-	+	+	-	-
Zhang et al. [53]; 2021	+	+	+	+	+	+	+	+	+

Supplementary Information D. Figures

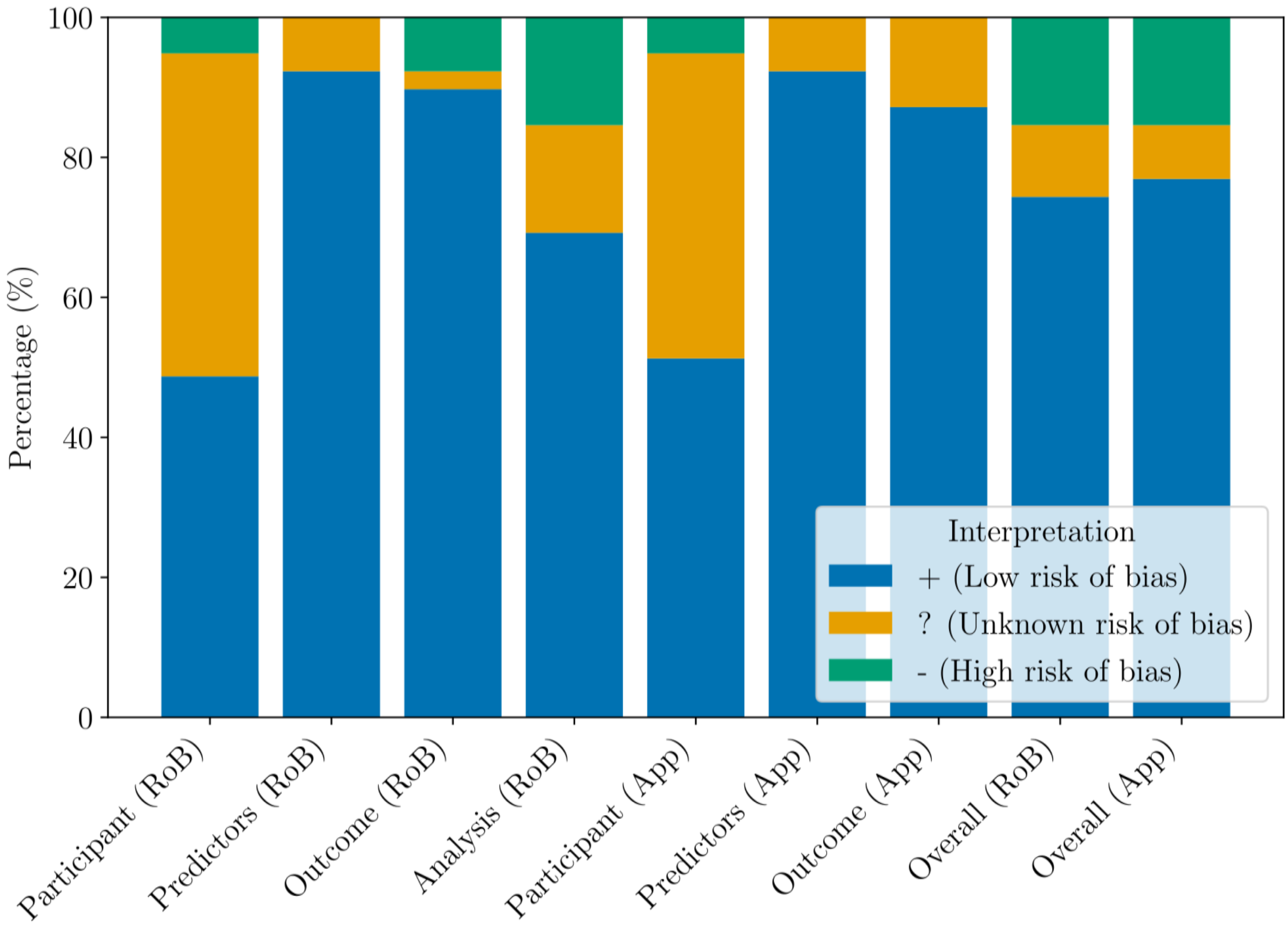

Figure S1 Prediction model study Risk Of Bias ASessment Tool (PROBAST). Results of studies meeting the inclusion criteria. Each bar represents the percentage of studies classified as having a low risk of bias or concern for applicability (+), an unknown risk of bias or concern for applicability (?), or a high risk of bias or concern for applicability (-). Risk of bias addresses impartial judgements of the selected studies, which systematically impact this review's analysis. Applicability refers to the extent to which the selected studies align with the PICO statement. The detailed analysis for each study is presented in Table S6. App, applicability; RoB, Risk of Bias.

Supplementary Information E.   PRISMA Checklists

Section and Topic	Item #	Checklist item	Location where item is reported (Page#)
**TITLE**			
Title	1	Identify the report as a systematic review.	1
**ABSTRACT**			
Abstract	2	See the PRISMA 2020 for Abstracts checklist.	Done
**INTRODUCTION**			
Rationale	3	Describe the rationale for the review in the context of existing knowledge.	4
Objectives	4	Provide an explicit statement of the objective(s) or question(s) the review addresses.	4
**METHODS**			
Eligibility criteria	5	Specify the inclusion and exclusion criteria for the review and how studies were grouped for the syntheses.	16–17
Information sources	6	Specify all databases, registers, websites, organisations, reference lists and other sources searched or consulted to identify studies. Specify the date when each source was last searched or consulted.	17
Search strategy	7	Present the full search strategies for all databases, registers and websites, including any filters and limits used.	17
Selection process	8	Specify the methods used to decide whether a study met the inclusion criteria of the review, including how many reviewers screened each record and each report retrieved, whether they worked independently, and if applicable, details of automation tools used in the process.	16–17
Data collection process	9	Specify the methods used to collect data from reports, including how many reviewers collected data from each report, whether they worked independently, any processes for obtaining or confirming data from study investigators, and if applicable, details of automation tools used in the process.	17
Data items	10a	List and define all outcomes for which data were sought. Specify whether all results that were compatible with each outcome domain in each study were sought (e.g. for all measures, time points, analyses), and if not, the methods used to decide which results to collect.	16–17
	10b	List and define all other variables for which data were sought (e.g. participant and intervention characteristics, funding sources). Describe any assumptions made about any missing or unclear information.	16–17
Study risk of bias assessment	11	Specify the methods used to assess risk of bias in the included studies, including details of the tool(s) used, how many reviewers assessed each study and whether they worked independently, and if applicable, details of automation tools used in the process.	18
Effect measures	12	Specify for each outcome the effect measure(s) (e.g. risk ratio, mean difference) used in the synthesis or presentation of results.	18
Synthesis methods	13a	Describe the processes used to decide which studies were eligible for each synthesis (e.g. tabulating the study intervention characteristics and comparing against the planned groups for each synthesis (item #5)).	17

Section and Topic	Item #	Checklist item	Location where item is reported (Page#)
	13b	Describe any methods required to prepare the data for presentation or synthesis, such as handling of missing summary statistics, or data conversions.	17
	13c	Describe any methods used to tabulate or visually display results of individual studies and syntheses.	17
	13d	Describe any methods used to synthesize results and provide a rationale for the choice(s). If meta-analysis was performed, describe the model(s), method(s) to identify the presence and extent of statistical heterogeneity, and software package(s) used.	18
	13e	Describe any methods used to explore possible causes of heterogeneity among study results (e.g. subgroup analysis, meta-regression).	17
	13f	Describe any sensitivity analyses conducted to assess robustness of the synthesized results.	18
Reporting bias assessment	14	Describe any methods used to assess risk of bias due to missing results in a synthesis (arising from reporting biases).	18
Certainty assessment	15	Describe any methods used to assess certainty (or confidence) in the body of evidence for an outcome.	17
**RESULTS**			
Study selection	16a	Describe the results of the search and selection process, from the number of records identified in the search to the number of studies included in the review, ideally using a flow diagram.	Fig 1
	16b	Cite studies that might appear to meet the inclusion criteria, but which were excluded, and explain why they were excluded.	10
Study characteristics	17	Cite each included study and present its characteristics.	Tables 1-3, Tables S1-S5
Risk of bias in studies	18	Present assessments of risk of bias for each included study.	Table S6
Results of individual studies	19	For all outcomes, present, for each study: (a) summary statistics for each group (where appropriate) and (b) an effect estimate and its precision (e.g. confidence/credible interval), ideally using structured tables or plots.	Tables 1-3, Tables S1-S5
Results of syntheses	20a	For each synthesis, briefly summarise the characteristics and risk of bias among contributing studies.	14; Fig S1
	20b	Present results of all statistical syntheses conducted. If meta-analysis was done, present for each the summary estimate and its precision (e.g. confidence/credible interval) and measures of statistical heterogeneity. If comparing groups, describe the direction of the effect.	Tables 1-3, Tables S1-S5, Figs 3-6
	20c	Present results of all investigations of possible causes of heterogeneity among study results.	8 and 12
	20d	Present results of all sensitivity analyses conducted to assess the robustness of the synthesized results.	Figs 5 and 6
Reporting biases	21	Present assessments of risk of bias due to missing results (arising from reporting biases) for each synthesis assessed.	14; Fig S1

Section and Topic	Item #	Checklist item	Location where item is reported (Page#)
Certainty of evidence	22	Present assessments of certainty (or confidence) in the body of evidence for each outcome assessed.	14; Fig S1
**DISCUSSION**			
Discussion	23a	Provide a general interpretation of the results in the context of other evidence.	7–14
	23b	Discuss any limitations of the evidence included in the review.	14
	23c	Discuss any limitations of the review processes used.	14–15
	23d	Discuss implications of the results for practice, policy, and future research.	14–16
**OTHER INFORMATION**			
Registration and protocol	24a	Provide registration information for the review, including register name and registration number, or state that the review was not registered.	4
	24b	Indicate where the review protocol can be accessed, or state that a protocol was not prepared.	4
	24c	Describe and explain any amendments to information provided at registration or in the protocol.	4
Support	25	Describe sources of financial or non-financial support for the review, and the role of the funders or sponsors in the review.	18–19
Competing interests	26	Declare any competing interests of review authors.	19
Availability of data, code and other materials	27	Report which of the following are publicly available and where they can be found: template data collection forms; data extracted from included studies; data used for all analyses; analytic code; any other materials used in the review.	18

From: Page MJ, McKenzie JE, Bossuyt PM, Boutron I, Hoffmann TC, Mulrow CD, et al. The PRISMA 2020 statement: an updated guideline for reporting systematic reviews. BMJ 2021;372:n71. doi: 10.1136/bmj.n71. This work is licensed under CC BY 4.0. To view a copy of this license, visit https://creativecommons.org/licenses/by/4.0/

Section and Topic	Item #	Checklist item	Reported (Yes/No)
**TITLE**			
Title	1	Identify the report as a systematic review.	Yes
**BACKGROUND**			
Objectives	2	Provide an explicit statement of the main objective(s) or question(s) the review addresses.	Yes
**METHODS**			
Eligibility criteria	3	Specify the inclusion and exclusion criteria for the review.	Yes
Information sources	4	Specify the information sources (e.g. databases, registers) used to identify studies and the date when each was last searched.	Yes
Risk of bias	5	Specify the methods used to assess risk of bias in the included studies.	Yes
Synthesis of results	6	Specify the methods used to present and synthesise results.	Yes
**RESULTS**			
Included studies	7	Give the total number of included studies and participants and summarise relevant characteristics of studies.	Yes
Synthesis of results	8	Present results for main outcomes, preferably indicating the number of included studies and participants for each. If meta-analysis was done, report the summary estimate and confidence/credible interval. If comparing groups, indicate the direction of the effect (i.e. which group is favoured).	Yes
**DISCUSSION**			
Limitations of evidence	9	Provide a brief summary of the limitations of the evidence included in the review (e.g. study risk of bias, inconsistency and imprecision).	Yes
Interpretation	10	Provide a general interpretation of the results and important implications.	Yes
**OTHER**			
Funding	11	Specify the primary source of funding for the review.	No
Registration	12	Provide the register name and registration number.	Yes

From: Page MJ, McKenzie JE, Bossuyt PM, Boutron I, Hoffmann TC, Mulrow CD, et al. The PRISMA 2020 statement: an updated guideline for reporting systematic reviews. BMJ 2021;372:n71. doi: 10.1136/bmj.n71. This work is licensed under CC BY 4.0. To view a copy of this license, visit https://creativecommons.org/licenses/by/4.0/

References

1 La Gerche, A. *et al.* Heart Failure With Preserved Ejection Fraction as an Exercise Deficiency Syndrome. *Journal of the American College of Cardiology* **80**, 1177–1191 (2022).

2 Costache, A.-D. *et al.* Cardiac Biomarkers in Sports Cardiology. *Journal of Cardiovascular Development and Disease* **9**, 453 (2022).

3 Biffi, A. *et al.* Premature ventricular beats in athletes: to detrain or not to detrain? *British Journal of Sports Medicine* **58**, 407–408 (2024).

4 McClean, G. *et al.* Electrical and structural adaptations of the paediatric athlete's heart: a systematic review with meta-analysis. *British Journal of Sports Medicine* **52**, 230–230 (2017).

5 Haykowsky, M. J., Samuel, T. J., Nelson, M. D. & La Gerche, A. Athlete's Heart: Is the Morganroth Hypothesis Obsolete? *Heart, Lung and Circulation* **27**, 1037–1041 (2018).

6 Heidbuchel, H. The athlete's heart is a proarrhythmic heart, and what that means for clinical decision making. *EP Europace* **20**, 1401–1411 (2017).

7 Drezner, J. A. *et al.* International criteria for electrocardiographic interpretation in athletes: Consensus statement. *British Journal of Sports Medicine* **51**, 704–731 (2017).

8 Boden, B. P., Breit, I., Beachler, J. A., Williams, A. & Mueller, F. O. Fatalities in High School and College Football Players. *AMERICAN JOURNAL OF SPORTS MEDICINE* **41**, 1108–1116–1108–1116 (2013).

9 Ha, F. J. *et al.* Sudden cardiac death related to physical exercise in the young: a nationwide cohort study of Australia. *Internal Medicine Journal* **53**, 497–502 (2022).

10 Marijon, E. *et al.* Characteristics and outcomes of sudden cardiac arrest during sports in women. *Circulation. Arrhythmia and electrophysiology* **6**, 1185–1191 (2013).

11 Maron, B. J., Haas, T. S., Ahluwalia, A. & Rutten-Ramos, S. C. Incidence of cardiovascular sudden deaths in Minnesota high school athletes. *Heart Rhythm* **10**, 374–377–374–377 (2013).

12 Risgaard, B. *et al.* Sports-related sudden cardiac death in a competitive and a noncompetitive athlete population aged 12 to 49 years: data from an unselected nationwide study in Denmark. *Heart Rhythm* **11**, 1673–1681–1673–1681 (2014).

13 Roberts, W. O. & Stovitz, S. D. Incidence of Sudden Cardiac Death in Minnesota High School Athletes 1993–2012 Screened With a Standardized Pre-Participation Evaluation. *Journal of the American College of Cardiology* **62**, 1298–1301–1298–1301 (2013).

14 Suarez-Mier, M. P., Aguilera, B., Mosquera, R. M. & Sanchez-de-Leon, M. S. Pathology of sudden death during recreational sports in Spain. *Forensic Sci Int* **226**, 188–196–188–196 (2013).

15 Wisten, A., Borjesson, M., Krantz, P. & Stattin, E.-L. Exercise related sudden cardiac death (SCD) in the young - Pre-mortal characterization of a Swedish nationwide cohort, showing a decline in SCD among athletes. *RESUSCITATION* **144**, 99–105–199–105 (2019).

16 Bohm, P. *et al.* Sports-Related Sudden Cardiac Arrest in Germany. *Can. J. Cardiol.* **37**, 105–112–105–112 (2021).

17 Bohm, P. *et al.* Sports-related sudden cardiac arrest in young adults. *Europace* **25**, 627–633–627–633 (2023).

18 Drezner, J. A. *et al.* Survival After Exercise-Related Sudden Cardiac Arrest in Young Athletes: Can We Do Better? *Sports Health: A Multidisciplinary Approach* **11**, 91–98–91–98 (2019).

19 Isern, C. B., Kramer-Johansen, J., Tjelmeland, I., Bahr, R. & Berge, H. M. A 3-year population-based study of exercise-related sudden cardiac arrest among 12- to 50-year-old Norwegians. *Scandinavian journal of medicine and science in sports* **33**, 1560–1569 (2023).

20 Sado, J. *et al.* Sports activity and paediatric out-of-hospital cardiac arrest at schools in Japan. *Resuscitation* **139**, 33–40 (2019).

21 Visanji, M. *et al.* Sports-Related Sudden Cardiac Arrest in Canada: Incidence and Survival. *Canadian Journal of Cardiology* **41**, 522–530 (2025).

22 Petek, B. J. *et al.* Sudden Cardiac Death in National Collegiate Athletic Association Athletes: A 20-Year Study. *Circulation* **149**, 80–90 (2024).

23 Peterson, D. F. *et al.* Aetiology and incidence of sudden cardiac arrest and death in young competitive athletes in the USA: a 4-year prospective study. *Br J Sports Med* **55**, 1196–1203–1196–1203 (2021).

24 Abousaber, I., El-Ghaish, H. & Abdallah, H. F. A novel explainable ECG classification with spatio-temporal transformers and hybrid loss optimization. *Biomed. Signal Process. Control* **110**, 108142–108142 (2025).

25 Ahn, S. *et al.* Artificial intelligence for predicting shockable rhythm during cardiopulmonary resuscitation: In-hospital setting. *Resuscitation* **202**, 110325–110325 (2024).

26 Ali, W., Abdullah, T. A. A., Zahid, M. S., Ahmed, A. A. & Abdulrab, H. Q. A. An Interpretable Model for Cardiac Arrhythmia Classification Using 1D CNN-GRU with Attention Mechanism. *Processes* **13** (2025).

27 Alqudah, A. M. & Alfraihat, A. A multimodal approach for cardiac signals classification using deep learning with explainable AI methods. *HEALTH INFORMATION SCIENCE AND SYSTEMS* **14** (2025).

28 Ayano, Y. M., Schwenker, F., Dufera, B. D., Debelee, T. G. & Ejegu, Y. G. Interpretable hybrid multichannel deep learning model for heart disease classification using 12-lead ECG signal. *IEEE Access* **12**, 94055–94080 (2024).

29 Cao, Y. *et al.* Detection and Localization of Myocardial Infarction Based on Multi-Scale ResNet and Attention Mechanism. *FRONTIERS IN PHYSIOLOGY* **13** (2022).

30 Chu, S. C., Li, Z., Pan, J. s., Tseng, K. & Kong, L. The Interpretable Graph Neural Network of ECG Beat Classification. *Journal of Internet Technology* **26**, 453–461 (2025).

31 Fiorina, L. *et al.* Near-term prediction of sustained ventricular arrhythmias applying artificial intelligence to single-lead ambulatory electrocardiogram. *European Heart Journal* **46**, 1998–2008 (2025).

32 Ganeshkumar, M., Ravi, V., S., V., E. A., G. & K. P., S. Explainable Deep Learning-Based Approach for Multilabel Classification of Electrocardiogram. *IEEE Transactions on Engineering Management* **70**, 2787–2799–2787–2799 (2023).

33 Habib, A., Karmakar, C. & Yearwood, J. Interpretability and Optimisation of Convolutional Neural Networks Based on Sinc-Convolution. *IEEE Journal of Biomedical and Health Informatics* **27**, 1758 – 1769–1758 – 1769 (2023).

34 Hammachi, R., Messaoudi, N. & Belkacem, S. ECG Beats Classification with Interpretability. *2022 International Conference of Advanced Technology in Electronic and Electrical Engineering (ICATEEE)*, 1–5–1–5 (2022).

35 Huang, Y. *et al.* ECG classification based on guided attention mechanism. *Comput. Methods Programs Biomed.* **257**, 108454–108454 (2024).
36 Jin, M.-N. *et al.* Association of Physical Activity With Primary Cardiac Arrest Risk in the General Population: A Nationwide Cohort Study of the Dose-Response Relationship. *Mayo Clinic Proceedings* **97**, 716 – 729–716 – 729 (2022).
37 Jo, Y.-Y. *et al.* Detection and classification of arrhythmia using an explainable deep learning model. *J Electrocardiol* **67**, 124–132–124–132 (2021).
38 Kolk, M. Z. H. *et al.* Dynamic prediction of malignant ventricular arrhythmias using neural networks in patients with an implantable cardioverter-defibrillator. *eBioMedicine* **99**, 104937–104937 (2024).
39 Kovalchuk, O., Barmak, O., Radiuk, P., Klymenko, L. & Krak, I. Towards transparent AI in medicine: ECG-based arrhythmia detection with explainable deep learning. *Technologies* **13**, 34–34 (2025).
40 Le, K. H. *et al.* LightX3ECG: A Lightweight and eXplainable Deep Learning System for 3-lead Electrocardiogram Classification. *Biomedical Signal Processing and Control* **85**, 104963 (2023).
41 Li, R., Aierken, Y., Xu, Y., Liu, J. & Tang, Y. Research on cross-dataset cardiac signal domain generalization and feature interpretability. *Sci Rep* **16**, 3138–3138 (2025).
42 Liu, X. *et al.* Advancing interpretable cardiac disease diagnosis via a transformer-convolutional hybrid network on electrocardiograms. *Engineering Applications of Artificial Intelligence* **152** (2025).
43 Lu, L. *et al.* Decoding 2.3 million ECGs: interpretable deep learning for advancing cardiovascular diagnosis and mortality risk stratification. *European Heart Journal-Digital Health* **5**, 247–259–247–259 (2024).
44 Mamun, A., Mia, N., Podder, T., Das, I. & Subah, J. Neuro-Causal Multimodal Transformer for Interpretable Arrhythmia Subtyping in Congenital and Pediatric ECG with Intracardiac Fusion. *2025 IEEE 2nd International Conference on Computing, Applications and Systems (COMPAS)*, 1–6 (2025).
45 Nankani, D. & Baruah, R. D. Ventricular Arrhythmia Classification and Interpretation Using Residual Neural Network with Guided Backpropagation. *2021 IEEE REGION 10 CONFERENCE (TENCON 2021)* **2021**, 574–579–574–579 (2021).
46 Neves, I. *et al.* Interpretable heartbeat classification using local model-agnostic explanations on ECGs. *Comput. Biol. Med.* **133**, 104393–104393 (2021).
47 Pasero, E., Casella, A. & Randazzo, V. Towards Explainable AI in Cardiac Diagnostic: Attention-Based Interpretation of Brugada Syndrome. *2025 IEEE International Conference on Artificial Intelligence for Learning and Optimization (ICoAILO)*, 1–6 (2025).
48 Potharaju, S. P. *et al.* Multi-resolution hybrid sliding window approach for ECG arrhythmia detection using deep learning models. *Discover Applied Sciences* **7** (2025).
49 Shylaja, R. & Kumari, L. V. R. ArrhythTransform: Multi-Head Attention-Based Transformer Encoder for Arrhythmia Classification. *ENGINEERING LETTERS* **33**, 4718–4730 (2025).
50 Tabatabaei, L., Yazdchi, M., Payan, H., Alamatsaz, N. & Nasimi, F. A lightweight hybrid CNN-LSTM explainable model for ECG-based arrhythmia detection. *Biomed. Signal Process. Control* **90**, 105884–105884 (2024).
51 Tao, R., Wang, L., Xiong, Y. & Zeng, Y.-R. IM-ECG: An interpretable framework for arrhythmia detection using multi-lead ECG. *Expert Systems with Applications* **237** (2024).
52 Xu, T., Sun, L., Subramani, S. & Wang, Y. Intelligent Interpretation and Classification of Multivariate Medical Time Series Based on Convolutional Neural Networks. *Health Information Science: 11th International Conference, HIS 2022, Virtual Event, October 28–30, 2022, Proceedings* **13705**, 291–302–291–302 (2022).
53 Zhang, D., Yang, S., Yuan, X. & Zhang, P. Interpretable deep learning for automatic diagnosis of 12-lead electrocardiogram. *iScience* **24** (2021).
54 Ai, W., Patel, N. D., Roop, P. S., Malik, A. & Trew, M. L. Cardiac Electrical Modeling for Closed-Loop Validation of Implantable Devices. *IEEE Transactions on Biomedical Engineering* **67**, 536–544–536–544 (2020).
55 Alaa, N. E., Lefraich, H. & El Malki, I. A second-generation computational modeling of cardiac electrophysiology: response of action potential to ionic concentration changes and metabolic inhibition. *Theoretical Biology and Medical Modelling* **11**, 46–46 (2014).
56 Biasi, N. & Tognetti, A. A computationally efficient dynamic model of human epicardial tissue. *PLoS ONE* **16**, e0259066–e0259066 (2021).
57 Bucelli, M. Mathematical and numerical modeling of the heart function: integrating electrophysiology, mechanics and fluid dynamics. (2022).
58 Chen, R., Cui, J., Li, S. & Hao, A. A Coupling Physics Model for Real-Time 4D Simulation of Cardiac Electromechanics. *Comput. Aided Des.* **175** (2024).
59 Collin, A., Chapelle, D. & Moireau, P. A Luenberger observer for reaction-diffusion models with front position data. *J. Comput. Phys.* **300**, 288–307–288–307 (2015).
60 Cusimano, N. & Gerardo-Giorda, L. A space-fractional Monodomain model for cardiac electrophysiology combining anisotropy and heterogeneity on realistic geometries. *J. Comput. Phys.* **362**, 409–424–409–424 (2018).
61 Del Corso, G., Verzicco, R. & Viola, F. A fast computational model for the electrophysiology of the whole human heart. *J. Comput. Phys.* **457**, 111084–111084 (2022).
62 Delestri, L. F. U. *et al.* Modelling of cardiac biventricular electromechanics with coronary blood flow to investigate the influence of coronary arterial motion on coronary haemodynamic. *Comput. Methods Prog. Biomed.* **267** (2025).
63 Elkaranshawy, H. A., Ali, A. M. E. & Abdelrazik, I. M. An effective heterogeneous whole-heart mathematical model of cardiac induction system with heart rate variability. *Proceedings of the Institution of Mechanical Engineers, Part H: Journal of Engineering in Medicine* **235**, 323–335–323–335 (2021).
64 Fedele, M. *et al.* A comprehensive and biophysically detailed computational model of the whole human heart electromechanics. *COMPUTER METHODS IN APPLIED MECHANICS AND ENGINEERING* **410**, 115983–115983 (2023).
65 Georgieva-Tsaneva, G. Mathematical Modeling Using Gaussian Functions and Chaotic Attractors: A Hybrid Approach for Realistic Representation of the Intrinsic Dynamics of Heartbeats. *AppliedMath* **5**, 172–172 (2025).
66 Gillette, K. *et al.* A Framework for the generation of digital twins of cardiac electrophysiology from clinical 12-leads ECGs. *Medical Image Analysis* **71**, 102080 (2021).
67 Hazim, A., Belhamadia, Y. & Dubljevic, S. Effects of mechano-electrical feedback on the onset of alternans: A computational study. *Chaos* **29** (2019).

68 Hurtado, D. E. & Kuhl, E. Computational modelling of electrocardiograms: repolarisation and T-wave polarity in the human heart. (2014).
69 Jackowska-Zduniak, B. Stochastic Models of the Slow/Fast Type of Atrioventricular Nodal Reentrant Tachycardia and Tachycardia with Conduction Aberration. *Int. J. Appl. Math. Comput. Sci.* **32**, 429–440–429–440 (2022).
70 Jacquemet, V. A simple analytical model of action potential duration profile in electrotonically-coupled cells. *MATHEMATICAL BIOSCIENCES* **272**, 92–99–92–99 (2016).
71 Jæger, K. H., Edwards, A. G., Giles, W. R. & Tveito, A. From Millimeters to Micrometers; Re-introducing Myocytes in Models of Cardiac Electrophysiology. *Frontiers in Physiology* **12**, 763584–763584 (2021).
72 Jæger, K. H. & Tveito, A. Efficient, cell-based simulations of cardiac electrophysiology; The Kirchhoff Network Model (KNM). *NPJ systems biology and applications* **9**, 25–25 (2023).
73 Johnston, B. M. & Johnston, P. R. A multi-electrode array and inversion technique for retrieving six conductivities from heart potential measurements. *Medical and Biological Engineering and Computing* **51**, 1295–1303–1295–1303 (2013).
74 Labarthe, S. *et al.* A bilayer model of human atria: mathematical background, construction, and assessment. *Europace* **16**, iv21–iv29–iv21–iv29 (2014).
75 Lee, Y., Cansız, B. & Kaliske, M. A multiphysical computational model of myocardial growth adopted to human pathological ventricular remodelling. *Comput. Mech.* **72**, 1215–1237–1215–1237 (2023).
76 Meisenzahl, C. *et al.* BOATMAP: Bayesian Optimization Active Targeting for Monomorphic Arrhythmia Pace-mapping. *Computers in Biology and Medicine* **182**, 109201—Article No.: 109201–Article No.: 109201–109201 (2024).
77 Moss, R., Wulfers, E. M., Schuler, S., Loewe, A. & Seemann, G. A Fully-Coupled Electro-Mechanical Whole-Heart Computational Model: Influence of Cardiac Contraction on the ECG. *Front Physiol* **12**, 778872–778872 (2021).
78 Pagani, S. *et al.* A Computational Study of the Electrophysiological Substrate in Patients Suffering From Atrial Fibrillation. *Front Physiol* **12**, 673612–673612 (2021).
79 Passow, A. & Tenderich, G. A mathematical approach to demonstrate R to T wave concordance of the human ECG. *Scientific Reports* **15** (2025).
80 Podziemski, P. & Zebrowski, J. J. A simple model of the right atrium of the human heart with the sinoatrial and atrioventricular nodes included. *J Clin Monit Comput* **27**, 481–498–481–498 (2013).
81 Quiroz-Juárez, M. A. *et al.* Cardiac Conduction Model for Generating 12 Lead ECG Signals With Realistic Heart Rate Dynamics. *IEEE Transactions on NanoBioscience* **17**, 525–532–525–532 (2018).
82 Rabinovitch, R. *et al.* Percolation and tortuosity in heart-like cells. *Scientific Reports* **11** (2021).
83 Romitti, G. S. *et al.* Implementation of a Cellular Automaton for efficient simulations of atrial arrhythmias. *Medical Image Analysis* **101** (2025).
84 Sabzpoushan, S. H. & Ghajarjazy, A. Simulating Notch-Dome Morphology of Action Potential of Ventricular Cell: How the Speeds of Positive and Negative Feedbacks on Transmembrane Voltage Can Influence the Health of a Cell? *BioMed Research International* **2020** (2020).
85 Salvador, M. *et al.* Electromechanical modeling of human ventricles with ischemic cardiomyopathy: numerical simulations in sinus rhythm and under arrhythmia. (2021).
86 Tenderini, R., Pagani, S., Quarteroni, A. & Deparis, S. PDE-Aware Deep Learning for Inverse Problems in Cardiac Electrophysiology. *SIAM J. Sci. Comput.* **44**, B605–B639–B605–B639 (2022).
87 Trovato, C. *et al.* Human Purkinje in silico model enables mechanistic investigations into automaticity and pro-arrhythmic abnormalities. *J Mol Cell Cardiol* **142**, 24–38–24–38 (2020).
88 Ugarte, J. P., Tobon, C., Lopes, A. M. & Tenreiro Machado, J. A. Atrial rotor dynamics under complex fractional order diffusion. (2018).
89 Wallman, M. & Sandberg, F. Characterisation of human AV-nodal properties using a network model. *Med Biol Eng Comput* **56**, 247–259–247–259 (2018).
90 Yang, C., Cao, Y., Li, P., Yang, Y. & Xiang, M. Computational Modeling of Cardiac Electrophysiology with Human Realistic Heart–Torso Model. *Bioengineering* **12**, 392–392 (2025).
91 Zappon, E. *et al.* An integrated heart-torso electromechanical model for the simulation of electrophysiological outputs accounting for myocardial deformation. *COMPUTER METHODS IN APPLIED MECHANICS AND ENGINEERING* **427** (2024).
